# Incidence of diabetes mellitus following hospitalisation for COVID‐19 in the United Kingdom: A prospective observational study

**DOI:** 10.1111/dom.16071

**Published:** 2024-11-20

**Authors:** Freya Tyrer, Safoora Gharibzadeh, Clare Gillies, Claire Lawson, Ash Routen, Nazrul Islam, Cameron Razieh, Francesco Zaccardi, Tom Yates, Melanie J. Davies, Christopher E. Brightling, James D. Chalmers, Annemarie B. Docherty, Omer Elneima, Rachael A. Evans, Neil J. Greening, Victoria C. Harris, Ewen M. Harrison, Ling‐Pei Ho, Alex Horsley, Linzy Houchen‐Wolloff, Olivia C. Leavy, Nazir I. Lone, Michael Marks, Hamish J. C. McAuley, Krisnah Poinasamy, Jennifer K. Quint, Betty Raman, Matthew Richardson, Ruth Saunders, Marco Sereno, Aarti Shikotra, Amish Singapuri, Louise V. Wain, Kamlesh Khunti, the PHOSP‐COVID Study Collaborative Group, C E Brightling, R A Evans, L V Wain, J D Chalmers, V C Harris, L P Ho, A Horsley, M Marks, K Poinasamy, B Raman, A Shikotra, A Singapuri, C E Brightling, R A Evans, L V Wain, R Dowling, C Edwardson, O Elneima, S Finney, N J Greening, B Hargadon, V C Harris, L Houchen‐Wolloff, O C Leavy, H J C McAuley, C Overton, T Plekhanova, R M Saunders, M Sereno, A Singapuri, A Shikotra, C Taylor, S Terry, C Tong, B Zhao, D Lomas, E Sapey, C Berry, C E Bolton, N Brunskill, E R Chilvers, R Djukanovic, Y Ellis, D Forton, N French, J George, N A Hanley, N Hart, L McGarvey, N Maskell, H McShane, M Parkes, D Peckham, P Pfeffer, A Sayer, A Sheikh, A A R Thompson, N Williams, C E Brightling, W Greenhalf, M G Semple, M Ashworth, H E Hardwick, L Lavelle‐Langham, W Reynolds, M Sereno, R M Saunders, A Singapuri, V Shaw, A Shikotra, B Venson, L V Wain, A B Docherty, E M Harrison, A Sheikh, J K Baillie, C E Brightling, L Daines, R Free, R A Evans, S Kerr, O C Leavy, N I Lone, H J C McAuley, R Pius, J Quint, M Richardson, M Sereno, M Thorpe, L V Wain, M Halling‐Brown, F Gleeson, J Jacob, S Neubauer, B Raman, S Siddiqui, J M Wild, S Aslani, P Jezzard, H Lamlum, W Lilaonitkul, E Tunnicliffe, J Willoughby, L V Wain, J K Baillie, H Baxendale, C E Brightling, M Brown, J D Chalmers, R A Evans, B Gooptu, W Greenhalf, H E Hardwick, R G Jenkins, D Jones, I Koychev, C Langenberg, A Lawrie, P L Molyneaux, A Shikotra, J Pearl, M Ralser, N Sattar, R M Saunders, J T Scott, T Shaw, D Thomas, D Wilkinson, L G Heaney, A De Soyza, D Adeloye, C E Brightling, J S Brown, J Busby, J D Chalmers, C Echevarria, L Daines, O Elneima, RA Evans, J Hurst, P Novotny, P Pfeffer, K Poinasamy, J Quint, I Rudan, E Sapey, M Shankar‐Hari, A Sheikh, S Siddiqui, S Walker, B Zheng, J R Geddes, M Hotopf, K Abel, R Ahmed, L Allan, C Armour, D Baguley, D Baldwin, C Ballard, K Bhui, G Breen, M Broome, T Brugha, E Bullmore, D Burn, F Callard, J Cavanagh, T Chalder, D Clark, A David, B Deakin, H Dobson, B Elliott, J Evans, R Francis, E Guthrie, P Harrison, M Henderson, A Hosseini, N Huneke, M Husain, T Jackson, I Jones, T Kabir, P Kitterick, A Korszun, I Koychev, J Kwan, A Lingford‐Hughes, P Mansoori, H McAllister‐Williams, K McIvor, L Milligan, R Morriss, E Mukaetova‐Ladinska, K Munro, A Nevado‐Holgado, T Nicholson, S Paddick, C Pariante, J Pimm, K Saunders, M Sharpe, G Simons, R Upthegrove, S Wessely, G P McCann, S Amoils, C Antoniades, A Banerjee, R Bell, A Bularga, C Berry, P Chowienczyk, J P Greenwood, A D Hughes, K Khunti, L Kingham, C Lawson, K Mangion, N L Mills, A J Moss, S Neubauer, B Raman, A N Sattar, C L Sudlow, M Toshner, P J M Openshaw, D Altmann, J K Baillie, R Batterham, H Baxendale, N Bishop, C E Brightling, P C Calder, R A Evans, J L Heeney, T Hussell, P Klenerman, J M Lord, P Moss, S L Rowland‐Jones, W Schwaeble, M G Semple, R S Thwaites, L Turtle, L V Wain, S Walmsley, D Wraith, M J Rowland, A Rostron, J K Baillie, B Connolly, A B Docherty, N I Lone, D F McAuley, D Parekh, A Rostron, J Simpson, C Summers, R G Jenkins, J Porter, R J Allen, R Aul, J K Baillie, S Barratt, P Beirne, J Blaikley, R C Chambers, N Chaudhuri, C Coleman, E Denneny, L Fabbri, P M George, M Gibbons, F Gleeson, B Gooptu, B Guillen Guio, I Hall, N A Hanley, L P Ho, E Hufton, J Jacob, I Jarrold, G Jenkins, S Johnson, M G Jones, S Jones, F Khan, P Mehta, J Mitchell, P L Molyneaux, J E Pearl, K Piper Hanley, K Poinasamy, J Quint, D Parekh, P Rivera‐Ortega, L C Saunders, M G Semple, J Simpson, D Smith, M Spears, L G Spencer, S Stanel, I Stewart, A A R Thompson, D Thickett, R Thwaites, L V Wain, S Walker, S Walsh, J M Wild, D G Wootton, L Wright, S Heller, M J Davies, H Atkins, S Bain, J Dennis, K Ismail, D Johnston, P Kar, K Khunti, C Langenberg, P McArdle, A McGovern, T Peto, J Petrie, E Robertson, N Sattar, K Shah, J Valabhji, B Young, L S Howard, Mark Toshner, C Berry, P Chowienczyk, D Lasserson, A Lawrie, O C Leavy, J Mitchell, J Newman, L Price, J Quint, A Reddy, J Rossdale, N Sattar, C Sudlow, A A R Thompson, J M Wild, M Wilkins, S J Singh, W D‐C Man, J M Lord, N J Greening, T Chalder, J T Scott, N Armstrong, E Baldry, M Baldwin, N Basu, M Beadsworth, L Bishop, C E Bolton, A Briggs, M Buch, G Carson, J Cavanagh, H Chinoy, E Daynes, S Defres, R A Evans, P Greenhaff, S Greenwood, M Harvie, M Husain, S MacDonald, A McArdle, H J C McAuley, A McMahon, M McNarry, G Mills, C Nolan, K O'Donnell, D Parekh, J Sargent, L Sigfrid, M Steiner, D Stensel, A L Tan, J Whitney, D Wilkinson, D Wilson, M Witham, D G Wootton, T Yates, D Thomas, N Brunskill, S Francis, S Greenwood, C Laing, K Bramham, P Chowdhury, A Frankel, L Lightstone, S McAdoo, K McCafferty, M Ostermann, N Selby, C Sharpe, M Willicombe, A Shaw, L Armstrong, B Hairsine, H Henson, C Kurasz, L Shenton, S Fairbairn, A Dell, N Hawkings, J Haworth, M Hoare, A Lucey, V Lewis, G Mallison, H Nassa, C Pennington, A Price, C Price, A Storrie, G Willis, S Young, P Pfeffer, K Chong‐James, C David, W Y James, C Manisty, A Martineau, O Zongo, A Sanderson, L G Heaney, C Armour, V Brown, T Craig, S Drain, B King, N Magee, D McAulay, E Major, L McGarvey, J McGinness, R Stone, A Haggar, A Bolger, F Davies, J Lewis, A Lloyd, R Manley, E McIvor, D Menzies, K Roberts, W Saxon, D Southern, C Subbe, V Whitehead, H El‐Taweel, J Dawson, L Robinson, D Saralaya, L Brear, K Regan, K Storton, J Fuld, A Bermperi, I Cruz, K Dempsey, A Elmer, H Jones, S Jose, S Marciniak, M Parkes, C Ribeiro, J Taylor, M Toshner, L Watson, J Weir McCall, J Worsley, R Sabit, L Broad, A Buttress, T Evans, M Haynes, L Jones, L Knibbs, A McQueen, C Oliver, K Paradowski, J Williams, E Harris, C Sampson, C Lynch, E Davies, C Evenden, A Hancock, K Hancock, M Rees, L Roche, N Stroud, T Thomas‐Woods, M Babores, J Bradley‐Potts, M Holland, N Keenan, S Shashaa, H Wassall, E Beranova, H Weston, T Cosier, L Austin, J Deery, T Hazelton, C Price, H Ramos, R Solly, S Turney, L Pearce, W McCormick, S Pugmire, W Stoker, A Wilson, N Hart, LA Aguilar Jimenez, G Arbane, S Betts, K Bisnauthsing, A Dewar, P Chowdhury, A Chiribiri, A Dewar, G Kaltsakas, H Kerslake, MM Magtoto, P Marino, LM Martinez, C O'Brien, M Ostermann, J Rossdale, TS Solano, E Wynn, N Williams, W Storrar, M Alvarez Corral, A Arias, E Bevan, D Griffin, J Martin, J Owen, S Payne, A Prabhu, A Reed, C Wrey Brown, C Lawson, T Burdett, J Featherstone, A Layton, C Mills, L Stephenson, N Easom, P Atkin, K Brindle, M G Crooks, K Drury, R Flockton, L Holdsworth, A Richards, D L Sykes, S Thackray‐Nocera, C Wright, K E Lewis, A Mohamed, G Ross, S Coetzee, K Davies, R Hughes, R Loosley, L O'Brien, Z Omar, H McGuinness, E Perkins, J Phipps, A Taylor, H Tench, R Wolf‐Roberts, L S Howard, O Kon, D C Thomas, S Anifowose, L Burden, E Calvelo, B Card, C Carr, E R Chilvers, D Copeland, P Cullinan, P Daly, L Evison, T Fayzan, H Gordon, S Haq, R G Jenkins, C King, K March, M Mariveles, L McLeavey, N Mohamed, S Moriera, U Munawar, J Nunag, U Nwanguma, L Orriss‐Dib, D O'Regan, A Ross, M Roy, E Russell, K Samuel, J Schronce, N Simpson, L Tarusan, C Wood, N Yasmin, R Reddy, A‐M Guerdette, M Hewitt, K Warwick, S White, A M Shah, C J Jolley, O Adeyemi, R Adrego, H Assefa‐Kebede, J Breeze, M Brown, S Byrne, T Chalder, A Chiribiri, P Dulawan, N Hart, A Hayday, A Hoare, A Knighton, M Malim, C O'Brien, S Patale, I Peralta, N Powell, A Ramos, K Shevket, F Speranza, A Te, P Beirne, A Ashworth, J Clarke, C Coupland, M Dalton, E Wade, C Favager, J Greenwood, J Glossop, L Hall, T Hardy, A Humphries, J Murira, D Peckham, S Plein, J Rangeley, G Saalmink, A L Tan, B Whittam, N Window, J Woods, G Coakley, D G Wootton, L Turtle, L Allerton, AM All, M Beadsworth, A Berridge, J Brown, S Cooper, A Cross, D Cuthbertson, S Defres, S L Dobson, J Earley, N French, W Greenhalf, H E Hardwick, K Hainey, J Hawkes, V Highett, S Kaprowska, G Kemp, AL Key, S Koprowska, L Lavelle‐Langham, N Lewis‐Burke, G Madzamba, F Malein, S Marsh, C Mears, L Melling, M J Noonan, L Poll, J Pratt, E Richardson, A Rowe, M G Semple, V Shaw, K A Tripp, B Vinson, L O Wajero, S A Williams‐Howard, J Wyles, S N Diwanji, P Papineni, S Gurram, S Quaid, G F Tiongson, E Watson, B Al‐Sheklly, A Horsley, C Avram, J Blaikely, M Buch, N Choudhury, D Faluyi, T Felton, T Gorsuch, N A Hanley, T Hussell, Z Kausar, C Miller, N Odell, R Osbourne, K Piper Hanley, K Radhakrishnan, S Stockdale, A De Soyza, C Echevarria, A Ayoub, J Brown, G Burns, G Davies, H Fisher, C Francis, A Greenhalgh, P Hogarth, J Hughes, K Jiwa, G Jones, G MacGowan, D Price, A Sayer, J Simpson, H Tedd, S Thomas, S West, M Witham, S Wright, A Young, M J McMahon, P Neill, D Anderson, H Bayes, C Berry, D Grieve, I B McInnes, N Basu, A Brown, A Dougherty, K Fallon, L Gilmour, K Mangion, A Morrow, K Scott, R Sykes, R Touyz, E K Sage, F Barrett, A Donaldson, M Patel, D Bell, A Brown, M Brown, R Hamil, K Leitch, L Macliver, J Quigley, A Smith, B Welsh, G Choudhury, J K Baillie, S Clohisey, A Deans, A B Docherty, J Furniss, E M Harrison, S Kelly, N I Lone, D E Newby, A Sheikh, J D Chalmers, D Connell, A Elliott, C Deas, J George, S Mohammed, J Rowland, A R Solstice, D Sutherland, C J Tee, N Maskell, D Arnold, S Barrett, H Adamali, A Dipper, S Dunn, A Morley, L Morrison, L Stadon, S Waterson, H Welch, B Jayaraman, T Light, C E Bolton, P Almeida, J Bonnington, M Chrystal, E Cox, C Dupont, P Greenhaff, A Gupta, L Howard, W Jang, S Linford, L Matthews, R Needham, A Nikolaidis, S Prosper, K Shaw, A K Thomas, L P Ho, N M Rahman, M Ainsworth, A Alamoudi, M Beggs, A Bates, A Bloss, A Burns, P Carter, M Cassar, K M Channon, J Chen, F Conneh, T Dong, R I Evans, E Fraser, X Fu, J R Geddes, F Gleeson, P Harrison, M Havinden‐Williams, P Jezzard, N Kanellakis, I Koychev, P Kurupati, X Li, E Lukaschuk, K McGlynn, H McShane, C Megson, K Motohashi, S Neubauer, D Nicoll, G Ogg, E Pacpaco, M Pavlides, Y Peng, N Petousi, J Propescu, N Rahman, B Raman, M J Rowland, K Saunders, M Sharpe, N Talbot, E Tunnicliffe, W D‐C Man, B Patel, R E Barker, D Cristiano, N Dormand, M Gummadi, S Kon, K Liyanage, C M Nolan, S Patel, O Polgar, P Shah, S J Singh, J A Walsh, J Hurst, H Jarvis, S Mandal, S Ahmad, S Brill, L Lim, D Matila, O Olaosebikan, C Singh, M Toshner, H Baxendale, L Garner, C Johnson, J Mackie, A Michael, J Pack, K Paques, H Parfrey, J Parmar, N Diar Bakerly, P Dark, D Evans, E Hardy, A Harvey, D Holgate, S Knight, N Mairs, N Majeed, L McMorrow, J Oxton, J Pendlebury, C Summersgill, R Ugwuoke, S Whittaker, W Matimba‐Mupaya, S Strong‐Sheldrake, S L Rowland‐Jones, A A R Thompson, J Bagshaw, M Begum, K Birchall, R Butcher, H Carborn, F Chan, K Chapman, Y Cheng, L Chetham, C Clark, Z Coburn, J Cole, M Dixon, A Fairman, J Finnigan, L Finnigan, H Foot, D Foote, A Ford, R Gregory, K Harrington, L Haslam, L Hesselden, J Hockridge, A Holbourn, B Holroyd‐Hind, L Holt, A Howell, E Hurditch, F Ilyas, C Jarman, A Lawrie, E Lee, J‐H Lee, R Lenagh, A Lye, I Macharia, M Marshall, A Mbuyisa, J McNeill, S Megson, J Meiring, L Milner, S Misra, H Newell, T Newman, C Norman, L Nwafor, D Pattenadk, M Plowright, J Porter, P Ravencroft, C Roddis, J Rodger, P Saunders, J Sidebottom, J Smith, L Smith, N Steele, G Stephens, R Stimpson, B Thamu, N Tinker, K Turner, H Turton, P Wade, S Walker, J Watson, I Wilson, A Zawia, R Aul, M Ali, A Dunleavy, D Forton, N Msimanga, M Mencias, T Samakomva, S Siddique, J Teixeira, V Tavoukjian, J Hutchinson, L Allsop, K Bennett, P Buckley, M Flynn, M Gill, C Goodwin, M Greatorex, H Gregory, C Heeley, L Holloway, M Holmes, J Kirk, W Lovegrove, TA Sewell, S Shelton, D Sissons, K Slack, S Smith, D Sowter, S Turner, V Whitworth, I Wynter, L Warburton, S Painter, J Tomlinson, C Vickers, T Wainwright, D Redwood, J Tilley, S Palmer, G A Davies, L Connor, A Cook, T Rees, F Thaivalappil, C Thomas, A Butt, M Coulding, H Jones, S Kilroy, J McCormick, J McIntosh, H Savill, V Turner, J Vere, E Fraile, J Ugoji, S S Kon, H Lota, G Landers, M Nasseri, S Portukhay, A Hormis, A Daniels, J Ingham, L Zeidan, M Chablani, L Osborne, M Marks, J S Brown, N Ahwireng, B Bang, D Basire, R C Chambers, A Checkley, R Evans, M Heightman, T Hillman, J Hurst, J Jacob, S Janes, R Jastrub, M Lipman, S Logan, D Lomas, M Merida Morillas, A Pakzad, H Plant, J C Porter, K Roy, E Wall, B Williams, M Xu, D Parekh, N Ahmad Haider, C Atkin, R Baggott, M Bates, A Botkai, A Casey, B Cooper, J Dasgin, K Draxlbauer, N Gautam, J Hazeldine, T Hiwot, S Holden, K Isaacs, T Jackson, S Johnson, V Kamwa, D Lewis, J M Lord, S Madathil, C McGhee, K Mcgee, A Neal, A Newton Cox, J Nyaboko, D Parekh, Z Peterkin, H Qureshi, B Rangelov, L Ratcliffe, E Sapey, J Short, T Soulsby, J Stockley, Z Suleiman, T Thompson, M Ventura, S Walder, C Welch, D Wilson, S Yasmin, K P Yip, P Beckett, C Dickens, U Nanda, C E Brightling, R A Evans, M Aljaroof, N Armstrong, H Arnold, H Aung, M Bakali, M Bakau, M Baldwin, M Bingham, M Bourne, C Bourne, N Brunskill, P Cairns, L Carr, A Charalambou, C Christie, M J Davies, S Diver, S Edwards, C Edwardson, O Elneima, H Evans, J Finch, S Glover, N Goodman, B Gootpu, N J Greening, K Hadley, P Haldar, B Hargadon, V C Harris, L Houchen‐Wolloff, W Ibrahim, L Ingram, K Khunti, A Lea, D Lee, G P McCann, H J C McAuley, P McCourt, T Mcnally, G Mills, A Moss, W Monteiro, M Pareek, S Parker, A Rowland, A Prickett, I N Qureshi, R Russell, N Samani, M Sereno, M Sharma, A Shikotra, S Siddiqui, A Singapuri, S J Singh, J Skeemer, M Soares, E Stringer, T Thornton, M Tobin, E Turner, L V Wain, T J C Ward, F Woodhead, J Wormleighton, T Yates, A Yousuf, M G Jones, C Childs, R Djukanovic, S Fletcher, M Harvey, E Marouzet, B Marshall, R Samuel, T Sass, T Wallis, H Wheeler, R Dharmagunawardena, E Bright, P Crisp, M Stern, A Wight, L Bailey, A Reddington, A Ashish, J Cooper, E Robinson, A Broadley, K Howard, L Barman, C Brookes, K Elliott, L Griffiths, Z Guy, D Ionita, H Redfearn, C Sarginson, A Turnbull, Y Ellis, M Marks, A Briggs, K Holmes, K Poinasamy, S Walker, M Halling‐Brown, G Breen, M Hotopf, K Lewis, N Williams

**Affiliations:** ^1^ Leicester Real World Evidence Unit, Diabetes Research Centre University of Leicester Leicester UK; ^2^ Centre for Ethnic Health, Diabetes Research Centre University of Leicester Leicester UK; ^3^ School of Primary Care, Population Sciences and Medical Education University of Southampton Southampton UK; ^4^ Leicester Diabetes Centre, Diabetes Research Centre University of Leicester Leicester UK; ^5^ The Institute of Lung Health, NIHR Leicester Biomedical Research Centre University of Leicester Leicester UK; ^6^ Ninewells Hospital and Medical School University of Dundee Dundee UK; ^7^ Centre for Medical Informatics, The Usher Institute University of Edinburgh Edinburgh UK; ^8^ MRC Human Immunology Unit University of Oxford Oxford UK; ^9^ Division of Infection, Immunity and Respiratory Medicine University of Manchester Manchester UK; ^10^ Centre for Exercise and Rehabilitation Science, NIHR Biomedical Research Centre University of Leicester Leicester UK; ^11^ Department of Population Health Sciences University of Leicester Leicester UK; ^12^ Department of Clinical Research London School of Hygiene & Tropical Medicine London UK; ^13^ Asthma and Lung UK London UK; ^14^ School of Public Health Imperial College London London UK; ^15^ Radcliffe Department of Medicine University of Oxford Oxford UK; ^16^ NIHR Leicester Biomedical Research Centre University of Leicester Leicester UK

**Keywords:** cohort study, population study, real‐world evidence, type 1 diabetes, type 2 diabetes

## Abstract

**Background:**

People hospitalised for coronavirus disease 2019 (COVID‐19) have elevated incidence of diabetes. However, it is unclear whether this is due to shared risk factors, confounding or stress hyperglycaemia in response to acute illness.

**Methods:**

We analysed a multicentre prospective cohort study (PHOSP‐COVID) of people ≥18 years discharged from NHS hospitals across the United Kingdom following COVID‐19. Individuals were included if they attended at least one research visit with a HbA1c measurement within 14 months of discharge and had no history of diabetes at baseline. The primary outcome was new onset diabetes (any type), as defined by a first glycated haemoglobin (HbA1c) measurement ≥6.5% (≥48 mmol/mol). Follow‐up was censored at the last HbA1c measurement. Age‐standardised incidence rates and incidence rate ratios (adjusted for age, sex, ethnicity, length of hospital stay, body mass index, smoking, physical activity, deprivation, hypertension, hyperlipidaemia/hypercholesterolaemia, intensive therapy unit admission, invasive mechanical ventilation, corticosteroid use and C‐reactive protein score) were calculated using Poisson regression. Incidence rates were compared with the control groups of published clinical trials in the United Kingdom by applying the same inclusion and exclusion criteria, where possible.

**Results:**

Incidence of diabetes was 91.4 per 1000 person‐years and was higher in South Asian (incidence rate ratios [IRR] = 3.60; 1.77, 7.32; *p* < 0.001) and Black ethnic groups (IRR = 2.36; 1.07, 5.21; *p* = 0.03) compared with White ethnic groups. When restricted to similar characteristics, the incidence rates were similar to those in UK clinical trials data.

**Conclusion:**

Diabetes incidence following hospitalisation for COVID‐19 is high, but it remains uncertain whether it is disproportionately higher than pre‐pandemic levels.

## 1 INTRODUCTION

Coronavirus disease 2019 (COVID‐19), an infectious disease caused by the severe acute respiratory syndrome coronavirus 2 (SARS‐CoV‐2),^1^ has had a devastating public health impact on mortality and morbidity worldwide.^2^ As of the December 2023, there have been almost 1.1 million hospitalisations for COVID‐19 in the United Kingdom.^3^ Around 10%–20% of people who have acute COVID‐19 infection have persistent symptoms (Long Covid) of fatigue, shortness of breath and cognitive dysfunction that continue beyond 12 weeks.^4^, ^5^, ^6^, ^7^ Among people hospitalised for COVID‐19, such persistent symptoms are highly prevalent, occurring in 50%–70% of individuals during the first 6–12 months after discharge,^2^, ^8^, ^9^ and are associated with a high risk of longer term health complications, most commonly cardiovascular, renal, respiratory or systemic conditions.^10^

One of the new‐onset conditions associated with COVID‐19, particularly in those hospitalised, is diabetes.^11^, ^12^, ^13^, ^14^, ^15^ The average excess risk of both Type 1 (T1DM) and Type 2 diabetes mellitus (T2DM) has been reported to be in the region of 30%–50%^16^, ^17^, ^18^, ^19^ but uncertainties remain.^20^ Much of the excess risk occurs in the first few weeks after COVID‐19 infection; however, elevated incidence has been found to endure beyond 1 year for T2DM, but not T1DM.^15^ The underlying biological mechanisms are complex but believed to due to hyperinflammation associated with the acute presentation of COVID‐19 and subsequent persistent inflammation that damages the organs and pancreatic beta‐cells to accelerate diabetes development.^18^, ^20^, ^21^, ^22^, ^23^ These metabolic effects appear to subside over time and do not appear to be related to obesity or pre‐diabetes status^15^ but are associated with inflammatory response, vaccination status and COVID‐19 severity.^14^, ^24^, ^25^

The majority of studies that estimate diabetes risk following COVID‐19 involve retrospective cohort studies, often using electronic health records, which means that the measurement of risk factors and diabetes may be less accurate and consistent than prospective cohort studies. Another common challenge relates to confounding as people with diabetes, including those that are undiagnosed prior to hospitalisation, are more likely to develop symptoms that require hospitalisation.^22^, ^23^ Diabetes and hospitalisation for COVID‐19 also share common risk factors, such as obesity, ethnicity, older ages and presence of co‐existing long‐term conditions.^26^ Moreover, glycaemic and metabolic changes as a response to stress (stress hyperglycaemia) are common in hospitalised individuals with acute illness^27^, ^28^ and can lead to an overestimation of new diabetes cases. Nonetheless, any potential increased incidence of diabetes in people who are hospitalised for COVID‐19 is a major public health concern as the burden of diabetes, in particular T2DM, is already large, affecting 462 million (6.3%) individuals globally and contributing to the top 10 leading causes of disability and mortality worldwide.^29^, ^30^, ^31^ The risk of future adverse health events following hospitalisation for COVID‐19 is also a priority area among those affected.^32^ With this in mind, the present study aimed to use prospective data to investigate the relationship between hospitalisation for COVID‐19 and incident diabetes.

## 2 MATERIALS AND METHODS

### 2.1 Study design and participants

The study followed the reporting of studies conducted using observational routinely‐collected health data (RECORD) checklist^33^ (Table S1). We used the PHOSP‐COVID cohort, a multicentre, prospective cohort study which has been described previously.^26^, ^34^ Briefly, the sample population comprised people aged 18 years and older who were discharged from 83 National Health Service (NHS) hospitals across the United Kingdom following admission to a medical assessment unit or ward for confirmed or clinician‐diagnosed COVID‐19 to 31 March 2021. As we required follow‐up data for this study, we included participants who consented to attend additional in‐person research visits (tier 2, 39 sites; Figure S1) within approximately 1‐year from discharge alongside routine clinical care.

### 2.2 Ethical approval

All study participants taking part in the PHOSP‐COVID study give written informed consent. The study has NHS ethics approval from the Leeds West Research Ethics Committee (20/YH/0225) and is registered on the ISRCTN Registry (ISRCTN10980107).

### 2.3 Procedures

#### 2.3.1 Participant selection

We included participants in the PHOSP‐COVID cohort if they attended additional in‐person research visits (i.e., tier 2 participants) at 2–7 months after discharge (5‐month visit) and/or at 10–14 months (1‐year visit). We included information on covariates available at baseline (i.e., at hospital discharge) that were conceptually related to both hospitalisation for COVID‐19 and incident diabetes: demographics, length of hospital stay, lifestyle (smoking, physical activity), comorbidities (hypertension, hyperlipidaemia/hypercholesterolaemia) and in‐hospital factors (intensive therapy unit [ITU] admission, invasive mechanical ventilation, corticosteroid use, C‐reactive protein). We excluded patients with missing baseline assessment dates (*n* = 2), a diagnosis of diabetes (self‐reported and/or through healthcare records) at baseline (*n* = 538), taking glucose‐lowering therapies at baseline (*n* = 41) or without HbA1c measurements post‐hospitalisation (*n* = 538). Figure S1 shows the population flow diagram.

#### 2.3.2 Outcome

The primary outcome for this analysis was new onset diabetes, defined as a first glycated haemoglobin (HbA1c) measurement ≥6.5% (≥48 mmol/mol). Follow‐up was censored at the last HbA1c measurement. Those without HbA1c measurements at post‐hospitalisation visits (*n* = 538) were excluded from the analysis.

#### 2.3.3 Statistical analysis

We described the characteristics of the population for potential confounders (i.e., conceptually related to both diabetes and COVID‐19 hospitalisations) by incident diabetes status, using means (SD; continuous covariates) and numbers (percentages; binary/categorical covariates).

Observed age‐standardised (10‐year groups, using mid‐year population statistics for 2021^35^) incidence rates for diabetes were calculated for males and females. Incidence rate ratios (IRRs) by age at admission (years), gender, ethnicity (White, South Asian, Black African/Caribbean, other/not stated [amalgamated for confidentiality]), length of hospital stay (<10, 10–<30, 30+ days), baseline body mass index (BMI; kg/m^2^), smoking status (smoker/ex‐smoker [amalgamated owing to small numbers]; non‐smoker); physical activity (from the General Practice Physical Activity Questionnaire [GPPAQ]^36^: inactive; moderately inactive; moderately active; active, not stated), quintile of multiple deprivation (IMD) (stratified into: 1 [most deprived]; 2 [second most deprived]; 3/4/unknown; 5 [least deprived]), hypertension, hyperlipidaemia/hypercholesterolaemia, ITU admission, invasive mechanical ventilation, corticosteroid (oral/intravenous [IV]) treatments and elevated serum C‐reactive protein score (>10.0 mg) were reported using Poisson regression with person‐time as a (log) offset (date of discharge from hospital [index date] to first diabetes/censoring date). As BMI was not available for all participants, we conducted a sensitivity analysis using BMI category (normal/underweight: BMI < 25 kg/m^2^ [<10 participants were underweight: BMI < 18.5 kg/m^2^]; overweight: BMI 25–<30 kg/m^2^; obese: BMI ≥ 30 kg/m^2^; and unknown BMI category). Both the World Health Organization and National Institute for Health and Care Excellence recommend a lower BMI cutoff of 27.5 kg/m^2^ among Asian populations^37^, ^38^ because they tend to have more centralised distribution of body fat^39^, ^40^ and need lower BMI and waist circumference to confer equivalent risk profiles.^41^ Therefore, we conducted a second sensitivity analysis using the lower BMI cutoff of 27.5 kg/m^2^ for obesity among South Asian participants.

To determine whether incident diabetes was disproportionately higher after hospitalisation for COVID‐19 using similar prospective designs with HbA1c follow‐up measurements, we compared our findings with the control groups of published clinical trials and prospective studies among adults (including non‐hospitalised patients) where the primary or secondary outcome was incident diabetes (or T2DM as the largest contributor to diabetes^31^), applying the same inclusion and exclusion criteria, where possible. Clinical trials and prospective cohort studies (primary research) published in the English language over the last 10 years (since 1 January 2010; search run 10 June 2024) were identified via a search of electronic health records (Medline [OVID platform]—Table S2 reports the search strategy). To replicate the PHOSP‐COVID cohort as far as possible, we only included studies carried out in the United Kingdom. All analyses were carried out in Stata v18.^42^

## 3 RESULTS

### 3.1 Demographic characteristics

From the initial sample of 7768 individuals, a total of 1426 were included in the study population (see Figure S1). Of these, 99 (6.9%) met the criteria (HbA1c ≥ 6.5%) for diabetes during follow‐up (mean HbA1c = 7.4% [range 6.5–15.8%]). Most individuals with diabetes (*n* = 68; 70%) were identified at their 5‐month visit; 33 (49%) of these individuals also had a 12‐month HbA1c measurement (i.e., 24/33 had persistent diabetes [HbA1c ≥ 6.5%]). Of those with both an HbA1c and CRP measurement at 5 months (*n* = 1223), a greater proportion of individuals with new‐onset diabetes had elevated serum C‐reactive protein scores (21% vs. 10%); this pattern was also observed at 12 months (22% vs. 9%). Among people identified with diabetes, 10% (*n* = 10/99) were recorded as taking glucose‐lowering medication between discharge and follow‐up. All individuals in the cohort were discharged from hospital between February 2020 and 31 March 2021.

In the total study population, mean age was 57.5 years (range 21–91), there were more males than females (59% vs. 41%) and most participants were white (77%; *n* = 1101), with 7.0% (*n* = 106) South Asian and 6% (*n* = 81) from Black African/Caribbean ethnic categories (Table 1). The other/unknown ethnic categories were most commonly mixed categories. Average (mean) BMI was in the obese range (≥30 kg/m^2^) and around half of participants (53%; *n* = 760) reported being physically inactive. Most individuals (66%; *n* = 877) were in hospital for <10 days. Overall corticosteroid use was high, with more than half (56%; *n* = 800) receiving oral or IV therapies; 93% (*n* = 1327) had elevated inflammation makers (C‐reactive protein >10.0 mg); 30% (*n* = 427) were treated in ITU; and 15% (*n* = 219) received invasive mechanical ventilation for COVID‐1related respiratory issues. At baseline, most individuals (*n* = 1410; 99%) had not yet been vaccinated (vaccine rollout was in December 2020 in the United Kingdom). A relatively low proportion of individuals reported feeling fully recovered from COVID‐19 at their 5‐month (*n* = 271; 19%) or 12‐month visit (*n* = 273; 19%); this was not substantially different for those who developed diabetes (5‐month visit: *n* = 14 [14%]; 12‐month visit: *n* = 21 [21%]).

**TABLE 1:** Baseline characteristics of study population, stratified by diabetes status at follow‐up.

Characteristics	No diabetes (*n* = 1327)			Diabetes (*n* = 99)		
	Number	Mean (SD)/percent	Range	Number	Mean (SD)/percent	Range
Mean follow‐up (years)	1327	0.8 (0.3)	0.1–1.2	99	0.7 (0.3)	0.2–1.2
Demographics						
Age at admission (years)	1327	57.4 (12.9)	21–91	99	58.3 (11.1)	28–83
Baseline BMI	978	31.7 (7.4)	17–79	72	32.7 (6.4)	22–55
Length of hospital stay, day						
<10	826	62.3		51	51.5	
10–<30	336	25.3		34	34.4	
30+	165	12.4		14	14.1	
Sex						
Male	784	59.1		54	54.5	
Female	543	40.9		45	45.5	
Ethnicity						
White	1041	78.4		60	60.6	
South Asian	92	6.9		14	14.1	
Black	71	5.4		10	10.1	
Other/not known	123	9.3		15	15.2	
Deprivation quintile						
1 (most deprived)	275	20.7		28	28.3	
2 (second most deprived)	296	22.3		23	23.2	
3/4/unknown	484	36.5		31	31.3	
5 (least deprived)	272	20.5		17	17.2	
Lifestyle						
Smoking						
Non‐smoker	740	55.8		63	63.6	
Ex‐smoker/smoker	587	44.2		36	36.4	
Physical activity						
Inactive	517	39.0		35	35.4	
Moderately inactive	192	14.5		16	16.2	
Moderately active	246	18.5		20	20.2	
Active	243	18.3		17	17.2	
Not stated	129	9.7		11	11.1	
Comorbidities^a^						
Hypertension						
Present	351	26.5		34	34.3	
Hyperlipidaemia/hypercholesterolaemia						
Present	192	14.5		15	15.2	
In‐hospital factors						
Corticosteroids (oral/IV)	741	55.8		59	59.6	
Intensive therapy unit admission	394	29.7		33	33.3	
Invasive mechanical ventilation	203	15.3		16	16.2	
Elevated C‐reactive protein score (>10.0 mg)	1232	92.8		95	96.0	

*Note*: Numbers <5 (or those that can be derived from missing rows) are not displayed or are amalgamated (most commonly ‘not stated’ with ‘no’) to preserve confidentiality.

Abbreviation: IV, intervenous.

^a^ Please see Table S2 for a full list of comorbidities.

### 3.2 Incidence of diabetes in PHOSP‐COVID cohort

During 1103.2 person‐years (PY) of observation, the age‐standardised incidence of diabetes was 91.4 per 1000 PY ([95% CI] 75.1, 11.2); 84.9 (65.0, 110.9) in males and 100.2 (74.9, 134.1) in females.

**FIGURE 1:**
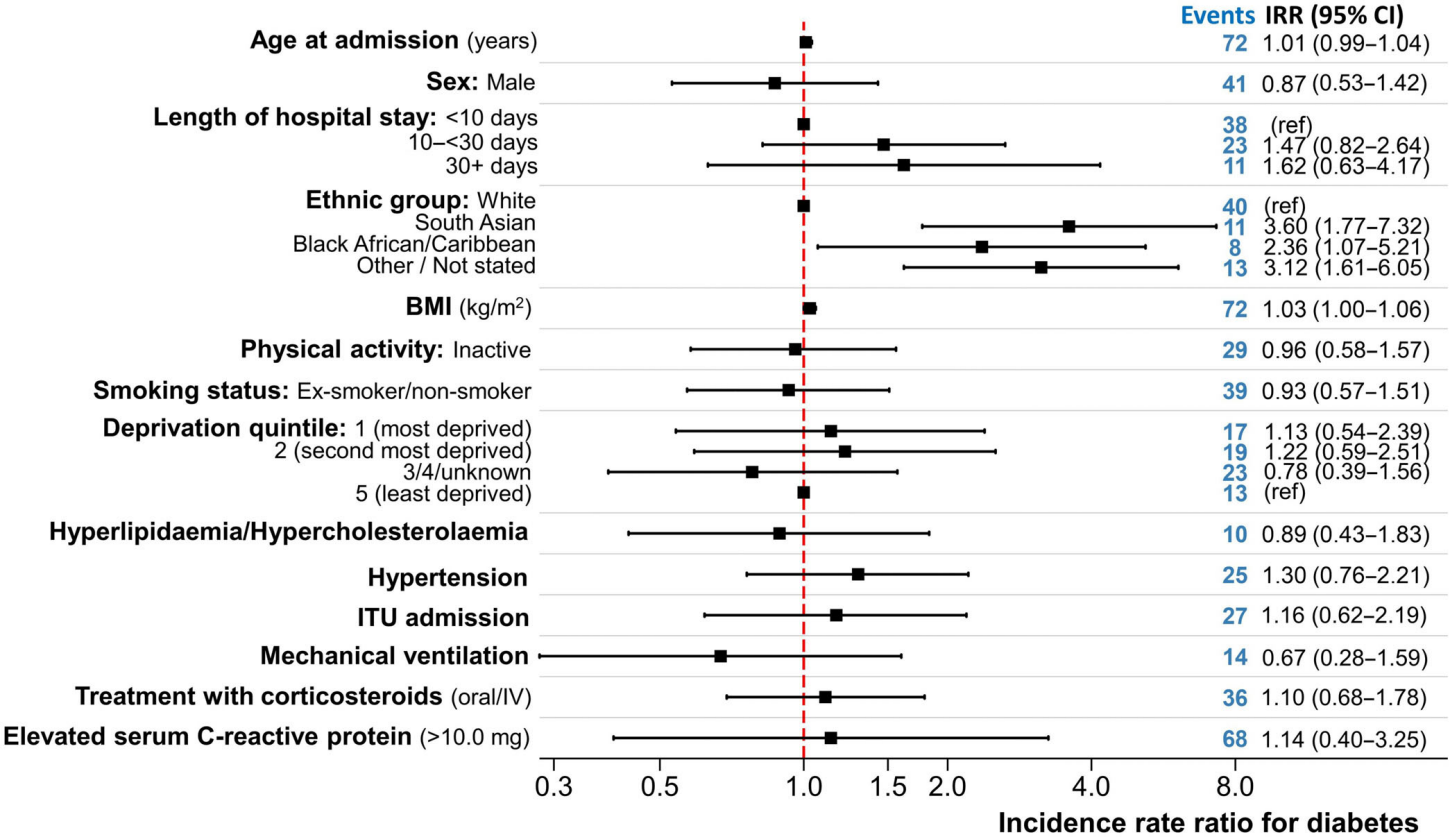
Incidence rate ratio for diabetes in PHOSP‐COVID sample (*n* = 1050). Patients with missing body mass index (BMI) were excluded from this analysis. The findings were repeated for all participants (using unknown BMI category as a separate category); see Figure S2. Estimates adjusted for all covariates shown in graph. CI, confidence interval; IRR, incidence rate ratios; ITU, intensive therapy unit.

The adjusted incidence rate ratios of diabetes by age, sex, ethnicity, length of hospital stay, BMI, smoking status, inactivity, deprivation status, presence of hypertension and hyperlipidaemia/hypercholesterolaemia, ITU admission, invasive mechanical ventilation, corticosteroid use and C‐reactive protein is shown in Figure 1. Compared with the White population, South Asian ethnic groups were around 3‐times more likely (IRR = 3.60 [95% CI: 1.77, 7.32]; *p* < 0.001) and Black ethnic groups around 2.4‐times more likely (IRR = 2.43 [1.07, 5.21]; *p* = 0.03) to have diabetes at follow‐up. We did not observe any independent effects of length of hospital stay, deprivation, smoking, self‐reported (GPPAQ) inactivity, hypertension, hyperlipidaemia/hypercholesterolaemia, ITU admission, invasive mechanical ventilation, corticosteroid use or C‐reactive protein. The sensitivity analysis using BMI category (including unknown) revealed marginally higher incidence rate ratios for South Asian individuals compared with white individuals (IRR = 3.62 [1.95, 6.74]; *p* < 0.001) (Figure S2). For Black African/Caribbean individuals, the rate of diabetes lowered to 2‐fold (compared with the White population) in the sensitivity analyses and was of borderline significance at the 5% level (IRR = 2.01 [1.00, 4.05]; *p* = 0.05). In this analysis, longer hospital stays (10–<30 vs. <10 days) were also associated with an increased risk of diabetes (IRR = 1.64 [1.02, 2.65]; *p* = 0.04). Similar findings were observed when BMI obesity threshold were lowered for South Asian participants (sensitivity analysis 2; Figure S3; IRR = 3.46 [1.87, 6.41]; *p* < 0.001 for South Asian vs. white ethnic groups).

### 3.3 Comparison with incidence rates from the literature

**FIGURE 2:**
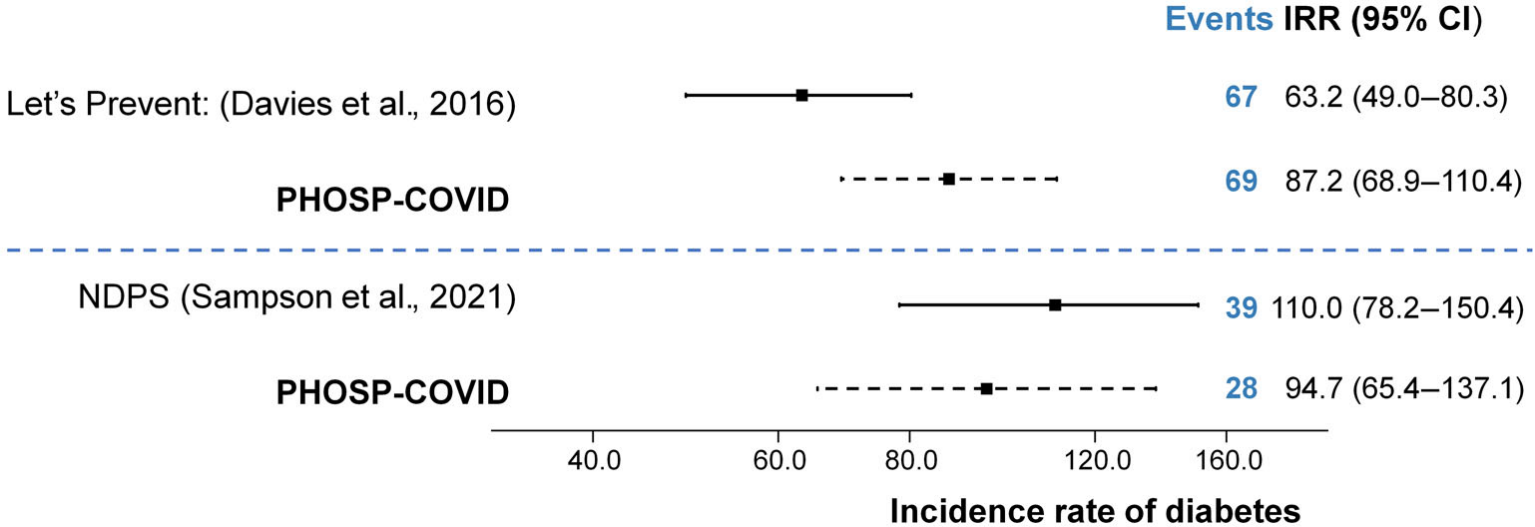
Comparison between PHOSP‐COVID incidence rate of diabetes (all) and clinical trials (Type 2 diabetes mellitus only). Please see Table S2 for details on inclusion/exclusion criteria for clinical trials and PHOSP‐COVID sample. CI, confidence interval; IRR, incidence rate ratios.

Of 67 articles identified from the Medline search, 19 studies were screened. The most common reason for rejecting studies was that the outcome encompassed self‐reported diabetes measures (*n* = 7; 37%). Most of the prospective cohort studies identified were based on UK Biobank data, incorporating a published algorithm to derive diabetes, based on linkage to primary and secondary care data^43^; these were rejected as HbA1c was not routinely collected as part of the study. Only two studies, both clinical trials, were identified as using comparable prospective methods (Figure S3 shows PRISMA^44^ flow diagram).^45^, ^46^ The trials are summarised in Table S2; both focused on incident T2DM and used control groups (for comparison) with pre‐diabetes (*n* = 433^45^) or non‐diabetic hyperglycaemia (*n* = 178^46^), The control populations in these trials received standard care or an information leaflet on risk factors for T2DM. Figure 2 illustrates how the diabetes incidence rates in PHOSP‐COVID differed from the trials when restricted to similar characteristics. The incidence rates in this PHOSP‐COVID cohort were similar: 87.2 [68.9, 110.4] versus 63.2 [49.0, 80.3]^45^ and 94.7 [65.4, 137.1] versus 110.0 [78.2, 150.4].^46^

## 4 DISCUSSION

In adults admitted to hospital with COVID‐19 in the United Kingdom, new‐onset diabetes occurred in 91.4 (75.1, 111.2) per 1000 person‐years. These findings are higher than estimates in non‐hospitalised individuals,^15^ but not markedly different from clinical trials of T2DM pre‐COVID when restricted to similar characteristics. In this PHOSP‐COVID cohort, people from South Asian (IRR: 3.6) and Black (IRR: 2.4) ethnic groups were more severely affected than White groups.

Overall, our findings are similar to previous research which has found that incident diabetes is high in hospitalised patients with COVID‐19.^15^, ^19^, ^47^ A recent systematic review and meta‐analysis of observational studies found an incidence rate of 15.5 (95% CI: 7.91, 25.64) per 1000 person‐years for all (i.e., including non‐hospitalised) COVID‐19 cases^48^ which is around 6‐times lower than the incidence rates observed in this PHOSP‐COVID population. For hospitalised COVID‐19, our findings are largely consistent with England‐wide findings of 99.5 per 1000 (vaccinated cohort) and 100.3 per 1000 (unvaccinated cohort) for T2DM and 1.7–13.5 per 1000 for T1DM, but are higher than the pre‐vaccination rates for T2DM of (44.5 per 1000).^15^ The findings are substantially higher than post‐discharge rates using the National Diabetes Audit (NDA) of 16.4 per 1000 population.^49^ Unlike this PHOSP‐COVID study, the NDA study excluded individuals that had diabetes prior to discharge and within 14 days post‐discharge. Previous evidence suggests that high glucose levels immediately following hospitalisation for COVID‐19 do not persist beyond the recovery stage.^15^ Our finding that more than one‐third (*n* = 9/33) individuals who met the criteria for diabetes at their 5‐month visit no longer met the required HbA1c threshold at their 12‐month visit, suggests that some of the diabetes cases that we identified resolved.

The comparison cohorts were chosen from non‐hospitalised prospective UK studies that tested HbA1c during follow‐up. One of the key differences between the PHOSP‐COVID population and those of the UK clinical trials we identified for comparison is that the trials were restricted to people who had elevated blood glucose levels (impaired fasting glycaemia or impaired glucose tolerance) but did not meet the criteria for diabetes. Previous studies of critically ill hospitalised patients (without diabetes at admission) suggest that around 47%–61% of patients exceed the upper limit of the normal glucose range because of acute or stress hyperglycaemia.^27^, ^28^ A further 48% of individuals treated with corticosteroids (56% of the PHOSP‐COVID cohort) present with steroid‐induced hyperglycaemia.^50^ Therefore, we would expect less than half of individuals in the PHOSP‐COVID cohort to have a normal glucose range at baseline. We included individuals who attended a 5‐month (range 2–7 months) and/or 12‐month (range 10–14 months) research visit after discharge from hospital, so we do not know how long HbA1c levels were elevated. Although associated with poorer outcomes^51^ and increased incidence of type 2 diabetes,^52^ around 73% of hyperglycaemia/diabetes resolves after alleviation of acute COVID‐19 illness,^15^ so we might expect the clinical trials to find higher incidence of diabetes than this hospitalised COVID‐19 cohort. However, we also need to balance this against the absence of a fully objective diabetes measure (diabetes was reported using self‐report and/or healthcare record) at baseline (an estimated 9% of adults presenting to hospital in the United Kingdom have undiagnosed diabetes^53^) and that the clinical trials were restricted to T2DM which accounts for only 90% of all diabetes cases.^54^ More persistent diabetes after COVID‐19 is hypothesised to be a result of prolonged inflammation that damages the organs and pancreatic beta‐cells.^18^, ^21^, ^22^, ^23^ This can also apply to other infections where insulin resistance (to facilitate the immune response) after recovery increases the susceptibility for diabetes.^55^ Previous research suggests that prevalence rates of diabetes are higher among people with pulmonary tuberculosis^56^ and human immunodeficiency virus (HIV).^57^ Large observational studies have also found higher incident rates of diabetes in people with COVID‐19 compared with influenza,^47^, ^58^ but have attributed this to greater clinical severity of COVID‐19 rather than the virus itself.^58^ We recommend further work in this area.

We found a higher relative incidence rate of diabetes in people from South Asian and black minority ethnic groups, but that this was not disproportionately greater from that expected in the general population. The incidence of diabetes among South Asian and Black African/Caribbean ethnic groups is around 2–4 times higher compared with White ethnic groups^59^, ^60^ which is consistent with the findings in this study. South Asian groups, in particular, are more likely to develop diabetes younger and with lower BMIs.^61^ The reasons for this are complex, but appear to driven by higher visceral adiposity leading to insulin resistance. It is also hypothesised that lean muscle mass, accentuated by hepatic fat accumulation, may reduce beta function and impair insulin action in South Asian populations.^62^ Our findings suggest that this may remain a concern among those hospitalised for COVID‐19, although we acknowledge that the sample size is small.

This study has the advantage of being population‐based, containing a large cohort of people hospitalised for COVID‐19 in the United Kingdom. Data were robust and prospectively collected, including objectively‐reported HbA1c measurements. However, we are unable to determine the type of incident diabetes in individuals studied. Thus far, research suggests that both T1DM and T2DM increase after COVID‐19^16^, ^17^, ^18^, ^19^; we are unable to provide further insight into this. Another limitation of the PHOSP‐COVID cohort is that it has a greater proportion of males and more severe COVID‐19 cases (i.e., requiring invasive ventilation)^63^ than those seen in the general UK hospital setting. Therefore, it may not be representative of COVID‐19 patients during that period as a whole. In particular, we were only able to follow‐up individuals who survived beyond discharge so may have missed some diabetes cases. We were also not able to look at people with newer variants of SARS‐CoV‐2, which may have had different effects on immune response. Similarly, returning participants for 1‐year visits may be different from those who do not return, even though their demographic characteristics have been found to be similar.^26^ Another key limitation is that comorbidities, including diabetes, were self‐reported and/or identified from healthcare records at baseline, so prone to response bias.

## 5 CONCLUSIONS

Diabetes incidence following hospitalisation for COVID‐19 is high, but there is uncertainty as to whether it is higher than pre‐pandemic levels in the absence of a representative control population. These patients need longer follow‐up to determine whether their diabetes goes into remission or has more aggressive progression as a result of inflammation post‐hospitalisation. Given that this hospitalised COVID‐19 cohort was typically obese, weight reduction strategies should be a consideration.

## AUTHOR CONTRIBUTIONS

The manuscript was initially drafted by Freya Tyrer and further developed by the writing committee. Melanie J. Davies, Safoora Gharibzadeh, Claire Lawson, Ash Routen, Tom Yates, Francesco Zaccardi and Kamlesh Khunti made substantial contributions to the conception and design of the work. Safoora Gharibzadeh made substantial contributions to the acquisition of data. Melanie J. Davies, Safoora Gharibzadeh, Claire Lawson, Ash Routen, Tom Yates, Francesco Zaccardi and Kamlesh Khunti made contributions to the analysis or interpretation of data for the work. Safoora Gharibzadeh verified the underlying data. All authors contributed to data interpretation and critical review and revision of the manuscript. All authors had full access to all the data in the study and had final responsibility for the decision to submit for publication.

## FUNDING INFORMATION

This work is independent research jointly funded by the National Institute for Health and Care Research (NIHR) and UK Research and Innovation (UKRI) (PHOSP‐COVID Post‐hospitalisation COVID‐19 study: a national consortium to understand and improve long‐term health outcomes, grant references: MR/V027859/1 and COV0319). The views expressed in this publication are those of the authors and not necessarily those of NIHR, The Department of Health and Social Care or UKRI. We also acknowledge funding from the Leicester National Institute for Health Research (NIHR) Biomedical Research Centre and Applied Research Collaboration (ARC) East Midlands.

## CONFLICT OF INTEREST STATEMENT

MJD has acted as consultant, advisory board member and speaker for Boehringer Ingelheim, Eli Lilly, Novo Nordisk and Sanofi, an advisory board member Pfizer, AstraZeneca, Zealand Pharma, Carmot/Roche and Medtronic and as a speaker for AstraZeneca and Amgen. She has received grants from AstraZeneca, Novo Nordisk, Boehringer Ingelheim, Janssen and Sanofi‐Aventis and Eli Lilly. TY has received investigator initiated funding from AstraZeneca for obesity related research. RAE declares that their institute was awarded a grant from UKRI/NIHR to complete this work; the author declares speaker fees from Boehringer Ingelheim and unpaid roles with European Respiratory Society Assembly 01.02 Pulmonary Rehabilitation secretary and American Thoracic Society Pulmonary Rehabilitation Assembly programme committee. JDC declares grants from AstraZeneca, Boehringer Ingelheim, Insmed, Novartis, Gilead Sciences, and Genentech; and consulting fees from AstraZeneca, Boehringer Ingelheim, Insmed, Novartis, Gilead Sciences, Chiesi, Zambon, and Genentech. LH‐W declares a grant from NIHR unrelated to the submitted work; acting as independent chair of the NIHR HTA Committee for Colour COPD trial; and membership of the American Thoracic Society Pulmonary Rehabilitation Assembly Web and Planning Committees. CEB has received grants and consultancy fees from 4D Pharma, Areteia, AstraZeneca, Chiesi, Genentech, GlaxoSmithKline, Mologic, Novartis, Regeneron Pharmaceuticals, Roche and Sanofi. British Lung Foundation, and Boehringer Ingleheim. AH declares that their institute was awarded a grant from UK Research and Innovation (UKRI) and NIHR to complete this work, and from NIHR Manchester Clinical Research Facility to support study delivery and NIHR Manchester Biomedical Research Centre (BRC) for personal funding and institutional payments to support grant‐funded research from NIHR, UK Medical Research Council (MRC), Cystic Fibrosis Trust, Cystic Fibrosis Foundation, North West Lung Centre Charity, and Moulton Trust; the author declares payment from Vertex Pharmaceuticals for educational presentation, participation on a clinical trials advisory board, and writing a review article. AH's non‐paid roles include chair of the Cystic Fibrosis Clinical Trials Accelerator Program, deputy chair of the NIHR Respiratory Translational Research Collaboration, and director of a university spin‐out company (Mi‐trial). NIL declares acting as director of research at the Intensive Care Society UK. PJMO declares co‐funding from MRC and GlaxoSmithKline (INFLAMMAGE), part of the EMINENT consortium to promote inflammation research; consulting fees from Janssen, Seqiris, and Valneva; payments for speaking from Janssen and Seqirus; and acting as member and vice‐chair of NERVTAG. LVW declares research funding unrelated to the submitted work from GlaxoSmithKline, Roche and Orion; consulting fees unrelated to the submitted work from Galapagos, GSK and Boehringer‐Ingelheim; travel support from Genentech; advisory board participation for Galapagos; and an associate editor role for the European Respiratory Journal. KK has acted as a consultant, speaker or received grants for investigator‐initiated studies for Astra Zeneca, Bayer, Novo Nordisk, Sanofi‐Aventis, Servier, Lilly and Merck Sharp & Dohme, Boehringer Ingelheim, Oramed Pharmaceuticals, Pfizer, Roche, Daiichi‐Sankyo, Applied Therapeutics, Embecta and Nestle Health Science. KK is supported by the National Institute for Health and Care Research (NIHR) Applied Research Collaboration East Midlands (ARC EM) and the NIHR Leicester Biomedical Research Centre (BRC). BR is funded by a Wellcome Career Development Award fellowship (302210/Z/23/Z). AShi, ASi, and MM declare that their institute was awarded a grant from UKRI/NIHR to complete this work. All other authors declare no competing interests

### PEER REVIEW

The peer review history for this article is available at https://www.webofscience.com/api/gateway/wos/peer-review/10.1111/dom.16071.

## ETHICS STATEMENT

All study participants taking part in the PHOSP‐COVID study give written informed consent. The study has NHS ethics approval from the Leeds West Research Ethics Committee (20/YH/0225) and is registered on the ISRCTN Registry (ISRCTN10980107).

## DISCLOSURE

Patient and public involvement has been integral to the PHOSP‐COVID study and consortium since conception. The original PHOSP PPI group was co‐chaired by NIHR Office for Clinical Research Infrastructure (NOCRI, Dr. Kate Holmes) and Asthma + Lung UK (Krisnah Poinasamy) with representation of over 10 relevant charities. Members of Long‐COVID support are closely involved and a Leicester BRC PPI group consisting of people with lived experience of a hospital admission for COVID‐19 (led by Linzy Houchen‐Wolloff). Patients and the public are embedded within the PHOSP infrastructure including our working groups, core management group, executive and steering groups. Patients were involved in the development of the clinical research study including the overarching aims, choice of outcomes, consent processes and the structure of the study visits. Patients reviewed all patient facing material. At the start of PHOSP, a joint patient and clinician research priority setting exercise was hosted by advisors from the James Lind Alliance to ensure co‐ownership of the direction of PHOSP‐COVID research questions. The data presented in the current manuscript starts to answer some of the co‐identified top 10 priorities.

## Supporting information


**Data S1.** Supporting information.

## Data Availability

The protocol, consent form, definition and derivation of clinical characteristics and outcomes, training materials, regulatory documents, information about requests for data access, and other relevant study materials are available online.
