# Supplementary material for: Incidence of diabetes mellitus following hospitalisation for COVID‐19 in the United Kingdom: A prospective observational study
Source: Diabetes Obes Metab. 2024 Nov 20;27(2):767–76. doi: 10.1111/dom.16071 (PMC11701205; doi:10.1111/dom.16071)
Supplement: Supplementary file 1 — Data S1. Supporting information. [file DOM-27-767-s001.docx]

**Incidence of Type 2 diabetes mellitus following hospitalisation for COVID-19 in the UK: a prospective observational study**

**Supplementary materials**

**Table S1. RECORD checklist (Reporting of studies conducted using observational routinely-collected data** **[1])**

|  | **Item No.** | **STROBE items** | **Location in manuscript where items are reported** | **RECORD items** | **Location in manuscript where items are reported** |
| --- | --- | --- | --- | --- | --- |
| **Title and Abstract** | | | | | |
|  | 1 | 1. Indicate the study’s design with a commonly used term in the title or the abstract 2. Provide in the abstract an informative and balanced summary of what was done and what was found | 🗹 Prospective study in **Title** and **Abstact**. Source of data (PHOSP-COVID) cited in **Abstract**.  🗹 See **Abstract** | RECORD 1.1: The type of data used should be specified in the title or abstract. When possible, the name of the databases used should be included.  RECORD 1.2: If applicable, the geographic region and timeframe within which the study took place should be reported in the title or abstract.  RECORD 1.3: If linkage between databases was conducted for the study, this should be clearly stated in the title or abstract. | 🗹 Source of data (PHOSP-COVID) cited in **Abstract**.  🗹 UK listed in **Abstract**  🗷 N/A |
| **Introduction** | | | | | |
| Background rationale | 2 | Explain the scientific background and rationale for the  investigation being reported | 🗹 See **Introduction** |  |  |
| Objectives | 3 | State specific objectives, including any prespecified hypotheses | 🗹 See last sentence of **Introduction** |  |  |
| **Methods** | | | | | |
| Study Design | 4 | Present key elements of study design early in the paper | 🗹 First sub-heading in **Material & Methods** [*study design and participants*] |  |  |
| Setting | 5 | Describe the setting, locations, and relevant dates, including  periods of recruitment, exposure, follow-up, and data collection | 🗹 Setting and location (first sub-heading in **Material & Methods**)  🗹 Observation period [recruitment & follow-up] (first sub-heading in **Material & Methods**)  🗹 First sub-heading in **Material & Methods** (i.e. hospitalisation for COVID-19) |  |  |
| Participants | 6 | 1. *Cohort study* - Give the eligibility criteria, and the sources and methods of selection of participants. Describe methods of follow-up   *Case-control study* - Give the eligibility criteria, and the sources and methods of case ascertainment and control selection. Give the rationale for the choice of cases and controls  *Cross-sectional study* - Give the eligibility criteria, and the sources and methods of selection of participants   1. *Cohort study* For matched studies, give matching criteria and number of exposed and unexposed   *Case-control study* - For matched studies, give matching criteria and the number of controls per case | 🗹 First sub-heading in **Material & Methods** [*study design and participants*]  N/A  N/A  N/A  N/A | RECORD 6.1: The methods of study population selection (such as codes or algorithms used to identify subjects) should be listed in detail. If this is not possible, an explanation should be provided.  RECORD 6.2: Any validation studies of the codes or algorithms used to select the population should be referenced. If validation was conducted for this study and not published elsewhere, detailed methods and results should be provided.  RECORD 6.3: If the study involved linkage of databases, consider use of a flow diagram or other graphical display to demonstrate the data linkage process, including the number of individuals with linked data at each stage. | 🗹 See Figure S1 (flowchart)  🗹 See covariates and Table 1 for coding and (limited) cleaning on the freetext - other comorbidities  🗹 See previous publications  🗹 Linkages not used but flowchart in Figure S1 describes data flow of population |
| Variables | 7 | Clearly define all outcomes, exposures, predictors, potential confounders, and effect modifiers. Give diagnostic criteria, if applicable. | 🗹 Outcome (diabetes) is described – including diagnostic criteria – in separate sub-heading (*Outcome*) in **Material & Methods**  All covariates used are described in **Material & Methods** | RECORD 7.1: A complete list of codes and algorithms used to classify exposures, outcomes, confounders, and effect modifiers should be provided. If these cannot be reported, an explanation should be provided. | 🗹 Diagnostic criteria from laboratory measurements for diabetes is provided. Other variables are provided in the text. Note – comorbidities were self-reported which is described and discussed as a limitation. |
| Data sources/ measurement | 8 | For each variable of interest, give sources of data and details of methods of assessment (measurement).  Describe comparability of assessment methods if there is more than one group | 🗹 See **Material & Methods** |  |  |
| Bias | 9 | Describe any efforts to address potential sources of bias | 🗹 People without HbA1c measurements removed (see **Material & Methods**) Right censoring at last HbA1c measure. Mortality prior to discharge discussed as a limitation (see **Discussion**) |  |  |
| Study size | 10 | Explain how the study size was arrived at | 🗹 All data from participating hospitals included. |  |  |
| Quantitative variables | 11 | Explain how quantitative variables were handled in the analyses. If applicable, describe  which groupings were chosen, and why | 🗹 See sub-heading *Statistical analysis* (**Material & Methods**) – ethnicity, body mass index, quintile of multiple deprivation and number of long-term conditions described. |  |  |
| Statistical methods | 12 | 1. Describe all statistical methods, including those used to control for confounding 2. Describe any methods used to examine subgroups and interactions 3. Explain how missing data were addressed 4. *Cohort study* - If applicable, explain how loss to follow-up was addressed   *Case-control study* - If applicable, explain how matching of cases and controls was addressed  *Cross-sectional study* - If applicable, describe analytical methods taking account of sampling strategy   1. Describe any sensitivity analyses | 🗹 See sub-heading *Statistical analysis* (**Material & Methods**)  🗹 Covariates described. Diabetes stratified in **Table 1**  🗹 Missing HbA1c data removed from analysis. Missing BMI data removed from IRRs (numbers given) but reanalysed as a separate subgroup (see sensitivity analysis)  🗹 Right censoring strategy described in **Material & Methods** (i.e. censored at last HbA1c measurement)  🗹 BMI group sensitivity analysis described and presented in supplementary materials |  |  |
| Data access and cleaning methods |  | - |  | RECORD 12.1: Authors should describe the extent to which the investigators had access to the database population used to create the study population.  RECORD 12.2: Authors should provide information on the data cleaning methods used in the study. | 🗹 PHOSP-COVID database and article of cohort description provided in **Material & Methods**  🗹 Minimal data cleaning required for comorbidities described. |
| Linkage |  | - |  | RECORD 12.3: State whether the study included person-level, institutional-level, or other data linkage across two or more databases. The methods of linkage and methods of linkage quality evaluation should be provided. | 🗷 N/A |
| **Results** | | | | | |
| Participants | 13 | 1. Report the numbers of individuals at each stage of the study (*e.g.*, numbers potentially eligible, examined for eligibility, confirmed eligible, included in the study, completing follow-up, and analysed) 2. Give reasons for non- participation at each stage. 3. Consider use of a flow diagram | 🗹 See data flow chart (supplementary **Figure S1**)  🗹 See data flow chart (supplementary **Figure S1**)  🗹 See data flow chart (supplementary **Figure S1**) | RECORD 13.1: Describe in detail the selection of the persons included in the study (*i.e.,* study population selection) including filtering based on data quality, data availability and linkage. The selection of included persons can be described in the text and/or by means of the study flow diagram. | 🗹 See data flow chart (supplementary **Figure S1**) |
| Descriptive data | 14 | 1. Give characteristics of study participants (*e.g.*, demographic, clinical, social) and information on exposures and potential confounders 2. Indicate the number of participants with missing data for each variable of interest 3. *Cohort study* - summarise follow-up time (*e.g.*, average and total amount) | 🗹 Baseline characteristics of the study population are shown in **Table 1**  🗹 See **Table 1** – please note: not all covariates described to preserve confidentiality  🗹 See **Table 1** and narrative (Results; *Incidence of diabetes*) with average follow-up and total person-years [1,103.2]) |  |  |
| Outcome data | 15 | *Cohort study* - Report numbers of outcome events or summary measures over time  *Case-control study* - Report numbers in each exposure category, or summary measures of exposure  *Cross-sectional study* - Report numbers of outcome events or summary measures | 🗹 Baseline characteristics (**Table 1**). Also presented separately for covariates under investigation in figures (**Figure 1,2**) |  |  |
| Main results | 16 | 1. Give unadjusted estimates and, if applicable, confounder- adjusted estimates and their precision (e.g., 95% confidence interval). Make clear which confounders were adjusted for and why they were included 2. Report category boundaries when continuous variables were categorized 3. If relevant, consider translating estimates of relative risk into absolute risk for a meaningful time period | 🗹 Confidence intervals were included and described. Rationale for potential confounder selection included in sub-heading *Statistical analyses* (**Material & Methods**)  🗹 number of comorbidities and BMI (sensitivity analysis)  🗹 Both relative (IRR) and absolute (incidence rates per 1000 PY) were reported for this analysis |  |  |
| Other analyses | 17 | Report other analyses done— e.g., analyses of subgroups and interactions, and sensitivity analyses | 🗹 See supplementary material for sensitivity analysis – also reported in narrative |  |  |
| **Discussion** | | | | | |
| Key results | 18 | Summarise key results with  reference to study objectives | 🗹 See **Results** |  |  |
| Limitations | 19 | Discuss limitations of the study, taking into account sources of potential bias or imprecision.  Discuss both direction and magnitude of any potential bias | 🗹 See last paragraph of **Discussion** | RECORD 19.1: Discuss the implications of using data that were not created or collected to answer the specific research question(s). Include discussion of misclassification bias, unmeasured confounding, missing data, and changing eligibility over time, as they pertain to the study being  reported. | 🗹 See last paragraph of **Discussion** |
| Interpretation | 20 | Give a cautious overall interpretation of results considering objectives, limitations, multiplicity of analyses, results from similar studies, and other relevant evidence | 🗹 See **Discussion** and **Conclusion** |  |  |
| Generalisability | 21 | Discuss the generalisability (external validity) of the study results | 🗹 See comparison with clinical trials (analysis – **Results**) and paragraphs 2–4 of **Discussion** |  |  |
| **Other Information** | | | | | |
| Funding | 22 | Give the source of funding and the role of the funders for the present study and, if applicable, for the original study on which the present article is based | 🗹 See **Funding** |  |  |
| Accessibility of protocol, raw data, and programming  code |  | .. |  | RECORD 22.1: Authors should provide information on how to access any supplemental information such as the study protocol, raw data, or  programming code. | 🗹 Study protocol is published and cited. Programming code is available on request |

**Table S2: Baseline comorbidities of the population by diabetes status**

| **Comorbidities** | | | | | | **No diabetes (n=1,327)** | | | **Diabetes (n=99)** | | |
| --- | --- | --- | --- | --- | --- | --- | --- | --- | --- | --- | --- |
|  | | | | | | **Number** | **Mean (SD)/**  **Percent** | **Range** | **Number** | **Mean (SD)/**  **Percent** | **Range** |
| **Total number** | | | | | 0 | 314 | 23.7 |  | 23 | 23.2 |  |
|  | | | | | 1 | 356 | 26.8 |  | 24 | 24.2 |  |
|  | | | | | 2+ | 657 | 49.5 |  | 52 | 52.5 |  |
| Cardiovascular disease^a^ | | | | |  | 159 | 12.0 |  | 15 | 15.2 |  |
| Hypertension |  | | | | | 351 | 26.5 |  | 34 | 34.3 |  |
| Hypercholesterolaemia/  dyslipidaemia | | | |  | | 192 | 14.5 |  | 15 | 15.2 |  |
| Stroke |  | | | | | 44 | 3.3 |  | - | - |  |
| Dementia |  | | | | | - | - |  | - | - |  |
| Depression or anxiety | | |  | | | 230 | 17.3 |  | 19 | 19.2 |  |
| Chronic fatigue, pain or fibromyalgia | | |  | | | 75 | 5.7 |  | - | - |  |
| Respiratory disorders^b^ | | |  | | | 357 | 26.9 |  | 28 | 28.3 |  |
| Chronic rheumatoid disorders^c^ | | |  | | | 204 | 15.4 |  | 18 | 18.2 |  |
| Gastrointestinal disorders^d^ | | | | |  | 266 | 20.1 |  | 18 | 18.2 |  |
| Thyroid disorders^e^ |  | | | | | 73 | 5.5 |  | 9 | 9.1 |  |
| Chronic kidney disease | |  | | | | 35 | 2.6 |  | - | - |  |
| Other metabolic disorders^f^ | | | |  | | 25 | 1.9 |  | - | - |  |
| Cancers^g^ |  | | | | | 40 | 3.0 |  | - | - |  |
| Infectious diseases^h^ |  | | | | | 97 | 98.0 |  | - | - |  |
| Other comorbidities^i^ |  | | | | | 344 | 25.9 |  | 20 | 20.2 |  |

Numbers <5 (or those that can be derived from missing rows) are not displayed or are combined with another category (most commonly ‘not stated’ with ‘no’) to preserve confidentiality; significance testing only applies categories with displayed information for both diabetes and no diabetes status

**^a^** cardiovascular disease: congestive heart failure, valvular heart disease, peripheral vascular disease

**^b^** respiratory disorders: asthma, chronic obstructive pulmonary disease (COPD), interstitial lung disease, bronchiectasis, obstructive sleep apnoea

**^c^** chronic rheumatoid disorders: connective tissue disease, rheumatoid arthritis, osteoarthritis, other chronic rheumatoid disorders

**^d^** gastrointestinal disorders: peptic ulcer, liver, gastro-oesophageal reflux, inflammatory bowel disease, irritable bowel syndrome, other gastrointestinal disorders

**^e^** thyroid disorders: hypothyroidism, hyperthyroidism

**^f^** other metabolic disorders: none of the participants had diabetes as specified in the design

**^g^** cancers: solid malignant tumours, leukaemia, lymphoma, other cancers

**^h^** infectious diseases: HIV, AIDS, Chronic viral hepatitis, mycobacterium tuberculosis, Other infectious diseases

^i^ freetext field after removing obvious errors (e.g. obesity was not considered a comorbidity)

**Figure S1: Data flow diagram**


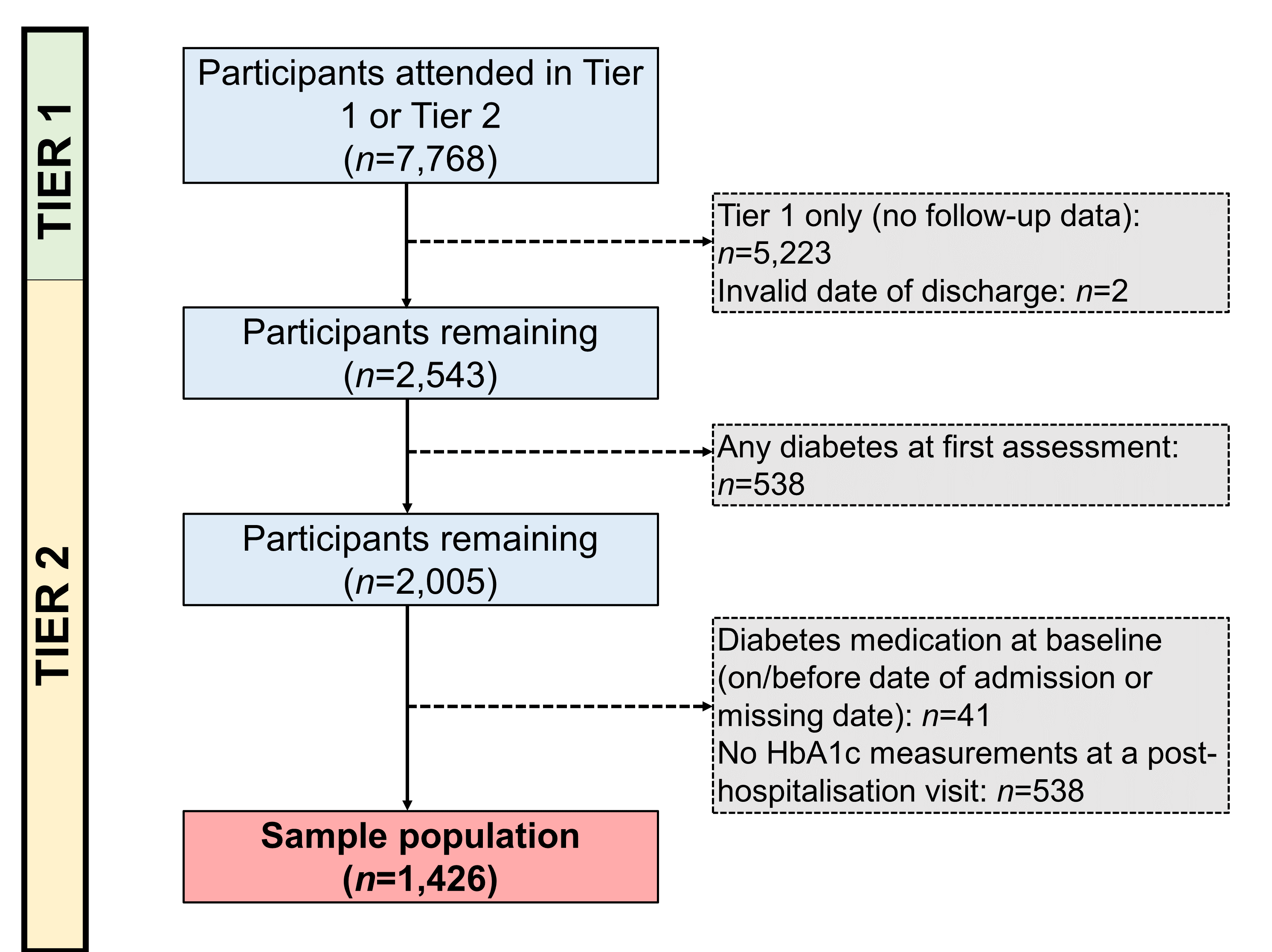


**Figure S2: Incidence rate ratio using body mass index (BMI) category (n=1,426)**


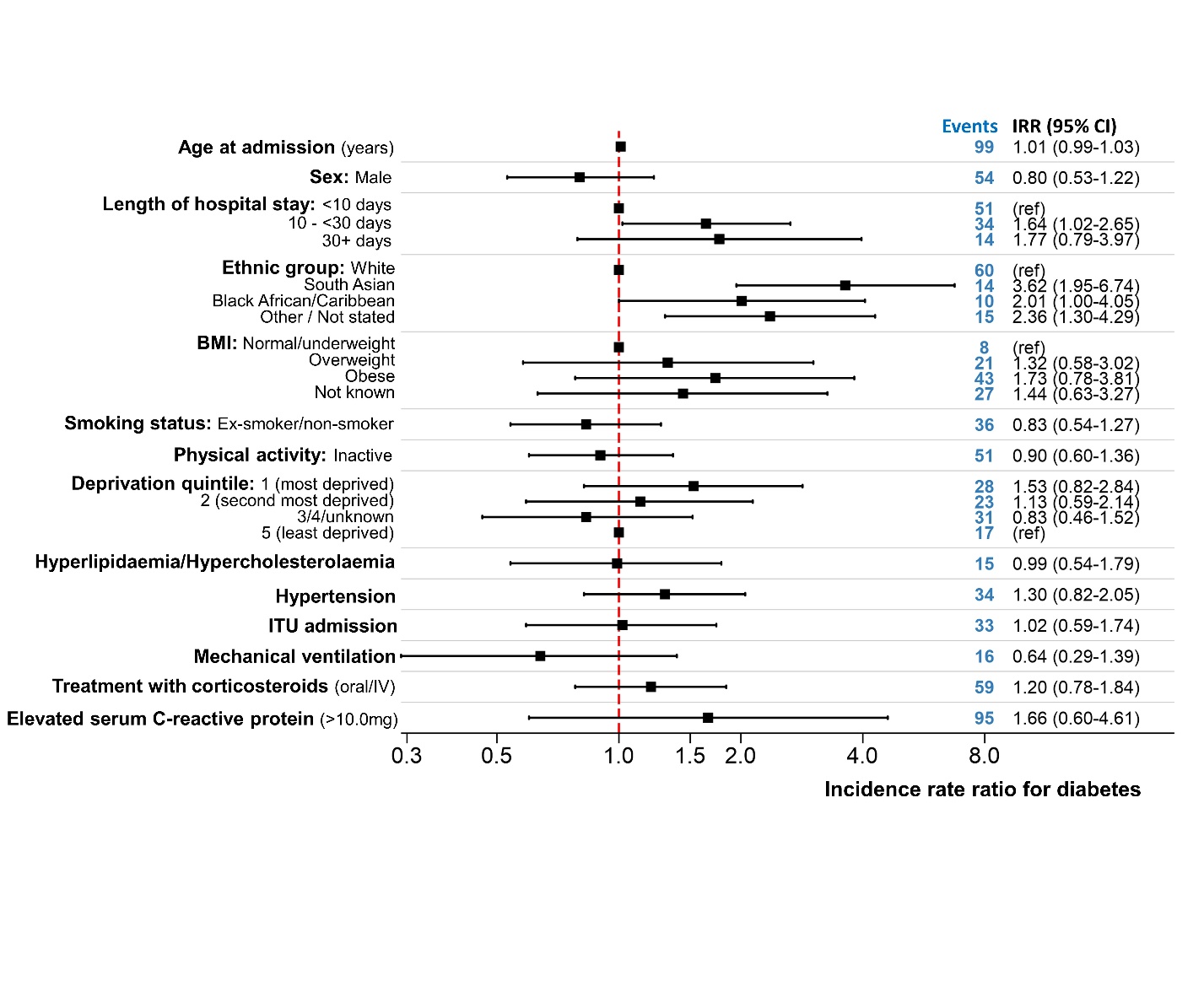


BMI: body mass index

**Figure S3: Incidence rate ratio using body mass index (BMI) category (n=1,426): lower threshold for South Asian participants**


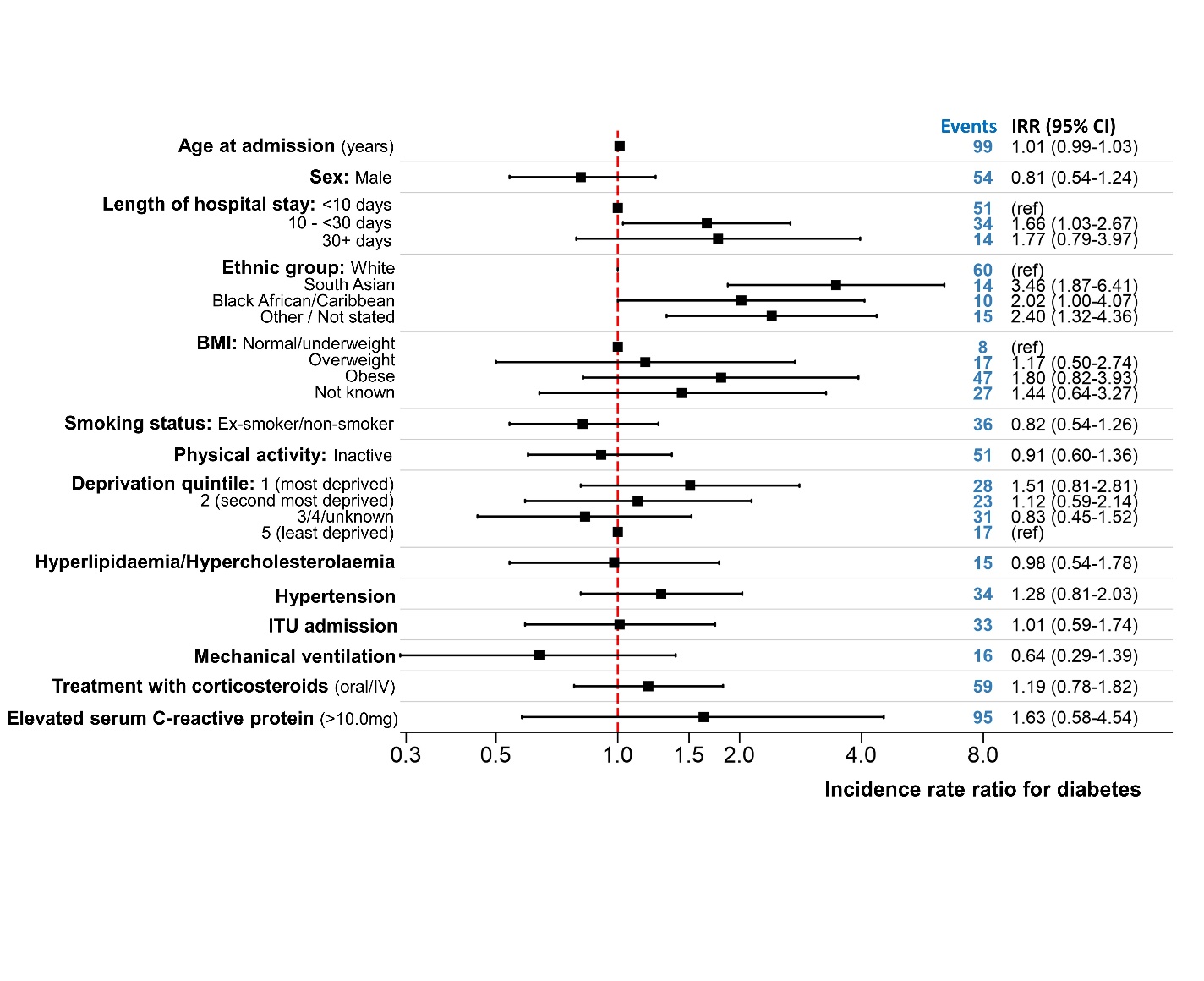


BMI: body mass index

**Table S3: Studies selected for comparison of diabetes incidence rates and analysis of PHOSP-COVID data for comparison**

| **ID** | **Author (date): *Study*** | **Country** | **Observation period** | **Inclusion criteria** | **Exclusion criteria** | **Control arm** | **How new-onset diabetes determined** | **Characteristics** | **Findings** |
| --- | --- | --- | --- | --- | --- | --- | --- | --- | --- |
| 1 | Davies et al. (2016) [2]  *Let’s Prevent* | UK | 2009–2014 | White: 40–75 yrs  S. Asian: 25–75yrs  Pre-diabetes (WHO 1999 criteria^a^) | Diabetes at baseline.  Unable to consent, pregnant/lactating, diabetes, terminal illness, needing interpreter for South Asian language other than named in the study | Information booklet on risk factors for diabetes | Pre-Jan 2013, WHO 1999 criteria for diabetes^b^  Post-Jan 2013: extended to also incorporate WHO 2011 criteria (HbA1c)^b^ | N=433  M: 64.2%  White: 84.3%  Mean age: 63.9yrs  Mean BMI: 33.1kg/m^2^ | 67/433 [1060 PY] developed T2DM    **Incidence rate: 63.2 (49.0, 80.3) per 1,000 PY^c^** |
|  | PHOSP-COVID comparison for Davies et al. (2016) | UK | 2020–2022 | White: 40–75 yrs  S. Asian: 25–75yrs | T2DM at baseline (for this subgroup analysis). Severe heart failure, severe ventricular systolic dysfunction, survival-limiting organ complications, long-term conditions related to coronary risk, recruitment in other studies | - | HbA1c≥6.5% (48mmol/mol) | N=1027  M: 58.7%  Mean age: 60.9yrs  Mean BMI: 32.0 kg/m^2^ | **Incidence rate: 87.2 (68.9, 110.4) per 1000 PY** |
| 2 | Sampson et al. 2021 [3] | UK | 2011–2019 | 40+ yrs  Non-diabetic hyperglycaemia: Paired HbA1c 6.0–<6.5% or FPG ≥5.6mmol/L (≥100mg/dL) –<7.0mmol/L (<126mg/dL) | Diabetes at baseline.  Inability to consent, pregnancy, lactating, self-reported chronic conditions, terminal illness/stage IV renal disease, heart failure, other health reasons defined by GP, self-reported medications: antipsychotics; high-dose oral steroids; active treatment for malignancy; ongoing dialysis | Usual care | Paired HbA1c≥6.5% or FPG≥126mg/dL | N=178  M:60.7%  Mean age: 65.3yrs;  Mean BMI: 31.2kg/m^2^ | 39/171 progressed to T2DM  **Incidence rate: 110.0 (78.2, 150.4) per 1,000 PY^c^** |
|  | PHOSP-COVID comparison for Sampson et al. (2021) | UK | 2020–2022 | 40+yrs | Diabetes at baseline.  Anyone with comorbidities with the exception of hypertension and hypercholesterolaemia/  hyperlipidaemia | - | HbA1c≥6.5% (48mmol/mol) | N=379  M:71.0%  Mean age: 57.2yrs  Mean BMI: 31.0kg/m^2^ | **Incidence rate: 94.7 (65.4,137.1) per 1,000 PY** |

BMI: body mass index; CHD: coronary heart disease; CKD: chronic kidney disease; DBP: diastolic blood pressure; F: females; F/H: family history; H/O: history of; IHD: ischaemic heart disease; M: males; yrs: years; PAD: peripheral arterial disease; PY: person-years; SBP: systolic blood pressure; SSRI: selective serotonin reuptake inhibitor

^a^ WHO criteria 1999 [4]

IGT: FPG <7.0mmol/L (<126mg/dL) and 2-h post glucose load ≥7.8mmol/L (≥140mg/dL) to <11.1mmol/L (<200mg/dL)

IFG: FPG ≥6.1mmol/L (≥110mg/dL) to <7.0mmol/L (<126mg/dL) and (if measured) 2-h plasma glucose <7.8mmol/L (<140mg/dL)

Diabetes: FPG ≥7.0mmol/dL (≥126mg/dL) or 2-h plasma glucose ≥11.1mmol/L (≥200mg/dL) [after a 75g oral glucose load]

^b^ WHO criteria 2011 [5]

WHO criteria for diabetes diagnosis extended to HbA1c≥6.5% (48mmol/L)

**^c^** Person-years derived from information in article; Poisson confidence intervals calculated.

**Table S4: Medline search strategy**

| 1 | ("risk of type 2 diabet*" or "incidence of type 2 diabet*" or "prevention of type 2 diabet*" or "new-onset type 2 diabet*" or "risk of diab*" or "incidence of diab*" or "prevention of diab*" or "new-onset diabet*").tw |
| --- | --- |
| 2 | ("risk of type 2 DM" or "incidence of type 2 DM" or "prevention of type 2 DM" or "new-onset type 2 DM" or "risk of DM" or "incidence of DM" or "prevention of DM" or "new-onset DM").tw |
| 3 | ("risk of type II diabet*" or "incidence of type II diabet*" or "prevention of type II diabet*" or "new-onset type II diabet*").tw |
| 4 | ("risk of type II DM" or "incidence of type II DM" or "prevention of type II DM" or "new-onset type II DM").tw |
| 5 | ("risk of type two diabet*" or "incidence of type two diabet*" or "prevention of type two diabet*" or "new-onset type two diabet*").tw. |
| 6 | ("risk of type 2 DM" or "incidence of type two DM" or "prevention of type two DM" or "new-onset type two DM").tw. |
| 7 | ("risk of T2DM" or "incidence of T2DM" or "prevention of T2DM" or "new-onset T2DM").tw |
| 8 | (("risk" or "incidence" or "prevention" or "new-onset") adj2 ("non-insulin" or "noninsulin" or "non insulin" or "NIDDM" or "DM2" or "diabetes" or "DM")).ti |
| 9 | or/1-8 |
| 10 | exp Clinical Trials as Topic/ |
| 11 | trial.ti. |
| 12 | "prospective".tw. |
| 13 | or/10-12 |
| 14 | 9 and 13 |
| 15 | Animals/ not Humans/ |
| 16 | 14 not 15 |
| 17 | ("systematic review" or "case-control" or "cohort study" or "metaanalysis" or "meta-an*" or "literature review*").tw. |
| 18 | ("protocol" or "pilot" or "feasibility" or “cohort profile”).ti. |
| 19 | or/17-18 |
| 20 | 16 not 19 |
| 21 | limit 20 to "review articles" |
| 22 | 20 not 21 |
| 23 | limit 22 to (english language and yr="2014 -Current") |
| 24 | ("UK" or "England" or "Scotland" or "Wales" or "Britain" or “Northern Ireland”).tw. |
| 25 | 23 and 24 |

**Figure S4: PRISMA** **[6] flow diagram**


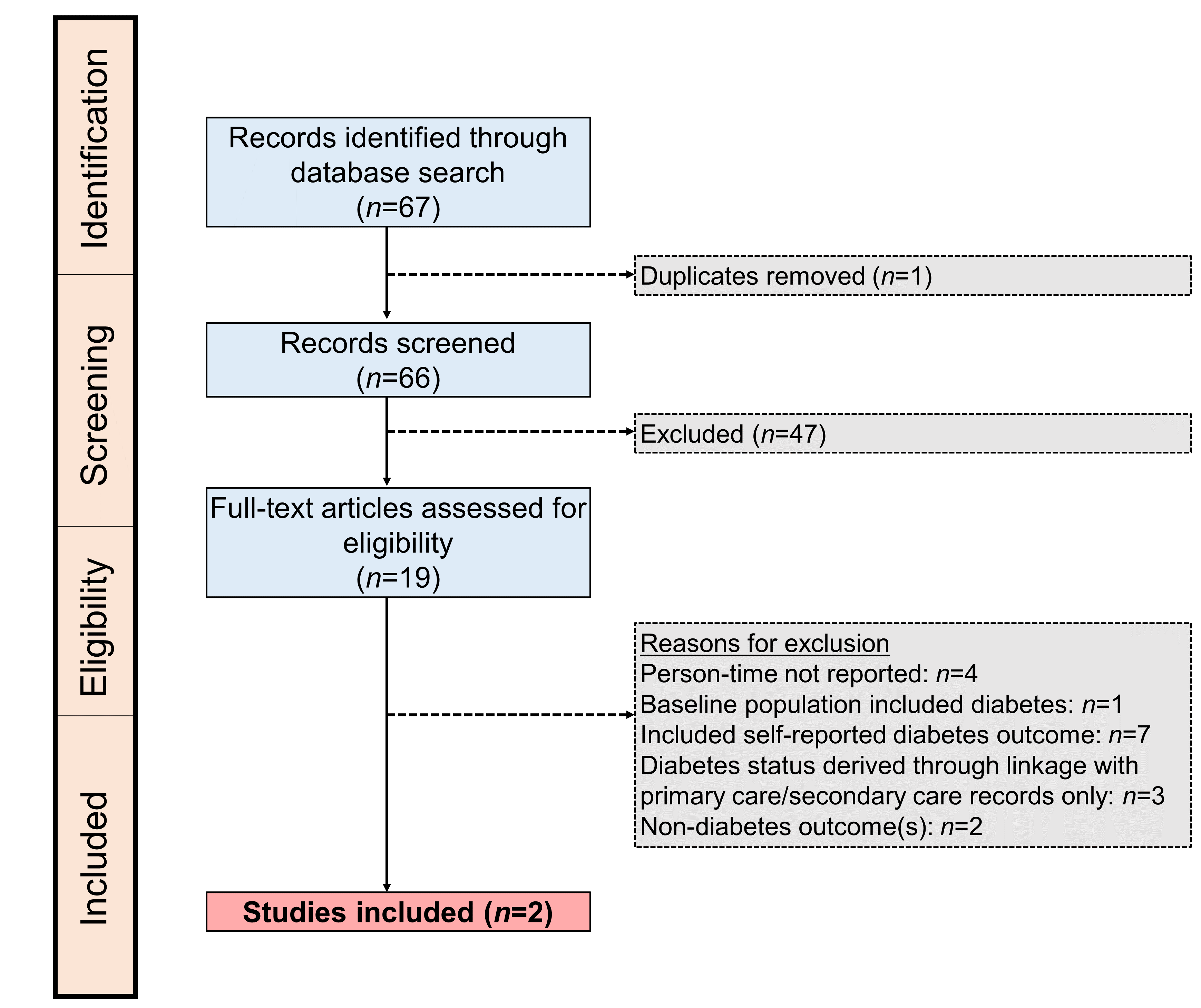


**Table S5: PHOSP-COVID study consortium**

| **PHOSP-COVID Collaborative Group** |
| --- |
| **Core Management Group** |
| *Chief Investigator* C E Brightling, *Members* R A Evans (Lead Co-I), L V Wain (Lead Co-I), J D Chalmers, V C Harris, L P Ho, A Horsley, M Marks, K Poinasamy, B Raman, A Shikotra, A Singapuri |
| **PHOSP-COVID Study Central Coordinating Team** |
| C E Brightling (Chief Investigator), R A Evans (*Lead Co-I*), L V Wain (*Lead Co-I*), R Dowling, C Edwardson, O Elneima, S Finney, N J Greening, B Hargadon, V C Harris, L Houchen--Wolloff, O C Leavy, H J C McAuley, C Overton, T Plekhanova, R M Saunders, M Sereno, A Singapuri, A Shikotra, C Taylor, S Terry, C Tong, B Zhao |
| **Steering Committee** |
| *Co-chairs* D Lomas, E Sapey*, Institution representatives* C Berry, C E Bolton, N Brunskill, E R Chilvers, R Djukanovic, Y Ellis, D Forton, N French, J George, N A Hanley, N Hart, L McGarvey, N Maskell, H McShane, M Parkes, D Peckham, P Pfeffer, A Sayer, A Sheikh, A A R Thompson, N Williams and core management group representation |
| **Executive Board** |
| *Chair* C E Brightling, representation from the core management group, each working group and platforms |
| **Platforms** |
| **Bioresource** |
| W Greenhalf (*Co-Lead*), M G Semple (*Co-Lead*), M Ashworth, H E Hardwick, L Lavelle-Langham, W Reynolds, M Sereno, R M Saunders, A Singapuri, V Shaw, A Shikotra, B Venson, L V Wain |
| **Data Hub** |
| A B Docherty (*Co-Lead*), E M Harrison (*Co-Lead*), A Sheikh (*Co-Lead*), J K Baillie, C E Brightling, L Daines, R Free, R A Evans, S Kerr, O C Leavy, N I Lone, H J C McAuley, R Pius, J Quint, M Richardson, M Sereno, M Thorpe, L V Wain |
| **Imaging Alliance** |
| M Halling-Brown (*Co-Lead*), F Gleeson (*Co-Lead*), J Jacob (*Co-Lead*), S Neubauer (*Co-Lead*) B Raman (*Co-Lead*) S Siddiqui (*Co-Lead*) J M Wild (*Co-Lead*), S Aslani, P Jezzard, H Lamlum, W Lilaonitkul, E Tunnicliffe, J Willoughby |
| **Omics** |
| L V Wain (*Co-Lead)*, J K Baillie (*Co-Lead*), H Baxendale, C E Brightling, M Brown, J D Chalmers, R A Evans, B Gooptu, W Greenhalf, H E Hardwick, R G Jenkins, D Jones, I Koychev, C Langenberg, A Lawrie, P L Molyneaux, A Shikotra, J Pearl, M Ralser, N Sattar, R M Saunders, J T Scott, T Shaw, D Thomas, D Wilkinson |
| **Working Groups** |
| **Airways** |
| L G Heaney (*Co-Lead*), A De Soyza (*Co-Lead*), D Adeloye, C E Brightling, J S Brown, J Busby, J D Chalmers, C Echevarria, L Daines, O Elneima, RA Evans, J Hurst, P Novotny, P Pfeffer, K Poinasamy, J Quint, I Rudan, E Sapey, M Shankar-Hari, A Sheikh, S Siddiqui, S Walker, B Zheng |
| **Brain** |
| J R Geddes (*Lead*), M Hotopf *(Co-Lead),* K Abel, R Ahmed, L Allan, C Armour, D Baguley, D Baldwin, C Ballard, K Bhui, G Breen, M Broome, T Brugha, E Bullmore, D Burn, F Callard, J Cavanagh, T Chalder, D Clark, A David, B Deakin, H Dobson, B Elliott, J Evans, R Francis, E Guthrie, P Harrison, M Henderson,  A Hosseini, N Huneke, M Husain, T Jackson, I Jones, T Kabir, P Kitterick, A Korszun, I Koychev, J Kwan, A Lingford-Hughes, P Mansoori, H McAllister-Williams, K McIvor, L Milligan, R Morriss, E Mukaetova-Ladinska, K Munro, A Nevado-Holgado, T Nicholson, S Paddick, C Pariante, J Pimm, K Saunders, M Sharpe, G Simons, R Upthegrove, S Wessely |
| **Cardiac** |
| G P McCann (*Lead*), S Amoils, C Antoniades, A Banerjee, R Bell, A Bularga, C Berry, P Chowienczyk, J P Greenwood, A D Hughes, K Khunti, L Kingham, C Lawson, K Mangion, N L Mills, A J Moss, S Neubauer, B Raman, A N Sattar, C L Sudlow, M Toshner, |
| **Immunology** |
| P J M Openshaw (*Lead*), D Altmann, J K Baillie, R Batterham, H Baxendale, N Bishop, C E Brightling, P C Calder, R A Evans, J L Heeney, T Hussell, P Klenerman, J M Lord, P Moss, S L Rowland-Jones, W Schwaeble, M G Semple, R S Thwaites, L Turtle, L V Wain, S Walmsley, D Wraith |
| **Intensive Care** |
| M J Rowland (*Lead*), A Rostron (*Co-Lead*), J K Baillie, B Connolly, A B Docherty, N I Lone, D F McAuley, D Parekh, A Rostron, J Simpson, C Summers |
| **Lung Fibrosis** |
| R G Jenkins (*Co-Lead*), J Porter (*Co-Lead*), R J Allen, R Aul, J K Baillie, S Barratt, P Beirne, J Blaikley, R C Chambers, N Chaudhuri, C Coleman, E Denneny, L Fabbri, P M George, M Gibbons, F Gleeson, B Gooptu, B Guillen Guio, I Hall, N A Hanley, L P Ho, E Hufton, J Jacob, I Jarrold, G Jenkins, S Johnson, M G Jones, S Jones, F Khan, P Mehta, J Mitchell, P L Molyneaux, J E Pearl, K Piper Hanley, K Poinasamy, J Quint, D Parekh, P Rivera-Ortega, L C Saunders, M G Semple, J Simpson, D Smith, M Spears, L G Spencer, S Stanel, I Stewart, A A R Thompson, D Thickett, R Thwaites, L V Wain, S Walker, S Walsh, J M Wild, D G Wootton, L Wright |
| **Metabolic** |
| S Heller (*Co-Lead*), M J Davies (*Co-Lead*), H Atkins, S Bain, J Dennis, K Ismail, D Johnston, P Kar, K Khunti, C Langenberg, P McArdle, A McGovern, T Peto, J Petrie, E Robertson, N Sattar, K Shah, J Valabhji, B Young |
| **Pulmonary and Systematic Vasculature** |
| L S Howard (*Co-Lead*), Mark Toshner (*Co-Lead*), C Berry, P Chowienczyk, D Lasserson, A Lawrie, O C Leavy, J Mitchell, J Newman, L Price, J Quint, A Reddy, J Rossdale, N Sattar, C Sudlow, A A R Thompson, J M Wild, M Wilkins |
| **Rehabilitation, Sarcopenia and Fatigue** |
| S J Singh (*Co-Lead*), W D-C Man (*Co-Lead*), J M Lord (*Co-Lead*), N J Greening (*Co-Lead*), T Chalder (*Co-Lead*), J T Scott (*Co-Lead*), N Armstrong, E Baldry, M Baldwin, N Basu, M Beadsworth, L Bishop, C E Bolton, A Briggs, M Buch, G Carson, J Cavanagh, H Chinoy, E Daynes, S Defres, R A Evans, P Greenhaff, S Greenwood, M Harvie, M Husain, S MacDonald, A McArdle, H J C McAuley, A McMahon, M McNarry, G Mills, C Nolan, K O’Donnell, D Parekh, Pimm, J Sargent, L Sigfrid, M Steiner, D Stensel, A L Tan, J Whitney, D Wilkinson, D Wilson, M Witham, D G Wootton, T Yates |
| **Renal** |
| D Thomas (*Lead*), N Brunskill (*Co-Lead*), S Francis (*Co-Lead*), S Greenwood (*Co-Lead*), C Laing (*Co-Lead*), K Bramham, P Chowdhury, A Frankel, L Lightstone, S McAdoo, K McCafferty, M Ostermann, N Selby, C Sharpe, M Willicombe |
| **Local Clinical Centre PHOSP-COVID trial staff** |
| (listed in alphabetical order) |
| **Airedale NHS Foundation Trust** |
| A Shaw (PI), L Armstrong, B Hairsine, H Henson, C Kurasz, L Shenton |
| **Aneurin Bevan University Health Board** |
| S Fairbairn (PI), A Dell, N Hawkings, J Haworth, M Hoare, A Lucey, V Lewis, G Mallison, H Nassa, C Pennington, A Price, C Price, A Storrie, G Willis, S Young |
| **Barts Health NHS Trust &** **Queen Mary University of London** |
| P Pfeffer (PI), K Chong-James, C David, W Y James, C Manisty, A Martineau, O Zongo |
| **Barnsley Hospital NHS Foundation Trust** |
| A Sanderson (PI) |
| **Belfast Health and Social Care Trust & Queen's University Belfast** |
| L G Heaney (PI), C Armour, V Brown, T Craig, S Drain, B King, N Magee, D McAulay, E Major, L McGarvey, J McGinness, R Stone |
| **Betsi Cadwaladr University Health Board** |
| A Haggar (PI), A Bolger, F Davies, J Lewis, A Lloyd, R Manley, E McIvor, D Menzies, K Roberts, W Saxon, D Southern, C Subbe, V Whitehead |
| **Borders General Hospital, NHS Borders** |
| H El-Taweel (PI), J Dawson, L Robinson |
| **Bradford Teaching Hospitals NHS Foundation Trust** |
| D Saralaya (PI), L Brear, K Regan, K Storton |
| **Cambridge University Hospitals NHS Foundation Trust, NIHR Cambridge Clinical Research Facility & University of Cambridge** |
| J Fuld (PI), A Bermperi, I Cruz, K Dempsey, A Elmer, H Jones, S Jose, S Marciniak, M Parkes, C Ribeiro, J Taylor, M Toshner, L Watson, J Weir McCall, J Worsley |
| **Cardiff and Vale University Health Board** |
| R Sabit (PI), L Broad, A Buttress, T Evans, M Haynes, L Jones, L Knibbs, A McQueen, C Oliver, K Paradowski, J Williams |
| **Chesterfield Royal Hospital NHS Trust** |
| E Harris (PI), C Sampson |
| **Cwm Taf Morgannwg University Health Board** |
| C Lynch (PI), E Davies, C Evenden , A Hancock, K Hancock, M Rees , L Roche, N Stroud, T Thomas-Woods |
| **East Cheshire NHS Trust** |
| M Babores (PI), J Bradley-Potts, M Holland, N Keenan, S Shashaa, H Wassall |
| **East Kent Hospitals University NHS Foundation Trust** |
| E Beranova (PI), H Weston (PI), T Cosier, L Austin, J Deery, T Hazelton, C Price, H Ramos, R Solly, S Turney |
| **Gateshead NHS Trust** |
| L Pearce (PI), W McCormick, S Pugmire, W Stoker, A Wilson |
| **Guy’s and St Thomas’ NHS Foundation Trust** |
| N Hart (PI), LA Aguilar Jimenez, G Arbane, S Betts, K Bisnauthsing, A Dewar, P Chowdhury, A Chiribiri, A Dewar, G Kaltsakas, H Kerslake, MM Magtoto, P Marino, LM Martinez, C O'Brien, M Ostermann, J Rossdale, TS Solano, E Wynn |
| **Hampshire Hospitals NHS Foundation Trust** |
| N Williams (PI), W Storrar (PI), M Alvarez Corral, A Arias, E Bevan, D Griffin, J Martin, J Owen, |
| S Payne, A Prabhu, A Reed, C Wrey Brown |
| **Harrogate and District NHD Foundation Trust** |
| C Lawson (PI), T Burdett, J Featherstone, A Layton, C Mills, L Stephenson, |
| **Hull University Teaching Hospitals NHS Trust & University of Hull** |
| N Easom (PI), P Atkin, K Brindle, M G Crooks, K Drury, R Flockton, L Holdsworth, A Richards, D L Sykes, S Thackray-Nocera, C Wright |
| **Hywel Dda University Health Board** |
| K E Lewis (PI), A Mohamed (PI), G Ross (PI), S Coetzee, K Davies, R Hughes, R Loosley, L O’Brien, Z Omar, H McGuinness, E Perkins, J Phipps, A Taylor, H Tench, R Wolf-Roberts |
| **Imperial College Healthcare NHS Trust & Imperial College London** |
| L S Howard (PI), O Kon (PI), D C Thomas (PI), S Anifowose, L Burden, E Calvelo, B Card, C Carr, E R Chilvers, D Copeland, P Cullinan, P Daly, L Evison, T Fayzan, H Gordon, S Haq, R G Jenkins, C King, K March, M Mariveles, L McLeavey, N Mohamed, S Moriera, U Munawar, J Nunag, U Nwanguma, L Orriss- Dib, D O'Regan, A Ross, M Roy, E Russell, K Samuel, J Schronce, N Simpson, L Tarusan, C Wood, N Yasmin |
| **Kettering General Hospital NHS Trust** |
| R Reddy (PI), A-M, Guerdette, M Hewitt, K Warwick, S White |
| **King’s College Hospital NHS Foundation Trust & Kings College London** |
| A M Shah (PI), C J Jolley (PI), O Adeyemi, R Adrego, H Assefa-Kebede, J Breeze, M Brown, S Byrne, T Chalder, A Chiribiri, P Dulawan, N Hart, A Hayday, A Hoare, A Knighton, M Malim, C O'Brien, S Patale, I Peralta, N Powell, A Ramos, K Shevket, F Speranza, A Te |
| **Leeds Teaching Hospitals & University of Leeds** |
| P Beirne (PI), A Ashworth, J Clarke, C Coupland, M Dalton, E Wade, C Favager, J Greenwood, J Glossop, L Hall, T Hardy, A Humphries, J Murira, D Peckham, S Plein, J Rangeley, G Saalmink, A L Tan, B Whittam, N Window, J Woods, |
| **Lewisham & Greenwich NHS Trust** |
| G Coakley (PI) |
| **Liverpool University Hospitals NHS Foundation Trust & University of Liverpool** |
| D G Wootton (PI), L Turtle (PI), L Allerton, AM All, M Beadsworth, A Berridge, J Brown, S Cooper, A Cross, D Cuthbertson, S Defres, S L Dobson, J Earley, N French, W Greenhalf, H E Hardwick, K Hainey, J Hawkes, V Highett, S Kaprowska, G Kemp, AL Key, S Koprowska, L Lavelle-Langham, N Lewis-Burke, G Madzamba, F Malein, S Marsh, C Mears, L Melling, M J Noonan, L Poll, J Pratt, E Richardson, A Rowe, M G Semple, V Shaw, K A Tripp, B Vinson, L O Wajero, S A Williams-Howard, J Wyles |
| **London North West University Healthcare NHS Trust** |
| S N Diwanji (PI), P Papineni (PI), S Gurram, S Quaid, G F Tiongson, E Watson |
| **Manchester University NHS Foundation Trust & University of Manchester** |
| B Al-Sheklly (PI), A Horsley (PI), C Avram, J Blaikely, M Buch, N Choudhury, D Faluyi, T Felton, T Gorsuch, N A Hanley, T Hussell, Z Kausar, C Miller, N Odell, R Osbourne, K Piper Hanley, K Radhakrishnan, S Stockdale |
| **Newcastle upon Tyne Hospitals NHS Foundation Trust & University of Newcastle** |
| A De Soyza (PI), C Echevarria (PI), A Ayoub, J Brown, G Burns, G Davies, H Fisher, C Francis, A Greenhalgh, P Hogarth, J Hughes, K Jiwa, G Jones, G MacGowan, D Price, A Sayer, J Simpson, H Tedd, S Thomas, S West, M Witham, S Wright, A Young |
| **NHS Dumfries and Galloway** |
| M J McMahon (PI), P Neill |
| **NHS Greater Glasgow and Clyde Health Board & University of Glasgow** |
| D Anderson (PI), H Bayes (PI), C Berry (PI), D Grieve (PI), I B McInnes (PI), N Basu, A Brown, A Dougherty, K Fallon, L Gilmour, K Mangion, A Morrow, K Scott, R Sykes, R Touyz |
| **NHS Highland** |
| E K Sage (PI), F Barrett, A Donaldson |
| **NHS Lanarkshire** |
| M Patel (PI), D Bell, A Brown, M Brown, R Hamil, K Leitch, L Macliver, J Quigley, A Smith, B Welsh |
| **NHS Lothian & University of Edinburgh** |
| G Choudhury (PI), J K Baillie, S Clohisey, A Deans, A B Docherty, J Furniss, E M Harrison, S Kelly, N I Lone, D E Newby, A Sheikh |
| **NHS Tayside & University of Dundee** |
| J D Chalmers (PI), D Connell, A Elliott, C Deas, J George, S Mohammed, J Rowland, A R Solstice, D Sutherland, C J Tee |
| **North Bristol NHS Trust & University of Bristol** |
| N Maskell (PI), D Arnold, S Barrett, H Adamali, A Dipper, S Dunn, A Morley, L Morrison, L Stadon, S Waterson, H Welch |
| **North Middlesex Hospital NHS Trust** |
| B Jayaraman (PI), T Light |
| **Nottingham University Hospitals NHS Trust & University of Nottingham** |
| C E Bolton (PI), P Almeida, J Bonnington, M Chrystal, E Cox, C Dupont, P Greenhaff, A Gupta, L Howard, W Jang, S Linford, L Matthews, R Needham, A Nikolaidis, S Prosper, K Shaw, A K Thomas |
| **Oxford University Hospitals NHS Foundation Trust & University of Oxford** |
| L P Ho (PI), N M Rahman (PI), M Ainsworth, A Alamoudi, M Beggs, A Bates, A Bloss, A Burns, P Carter, M Cassar, K M Channon, J Chen, F Conneh, T Dong, R I Evans, E Fraser, X Fu, J R Geddes, F Gleeson, P Harrison, M Havinden-Williams, P Jezzard, N Kanellakis, I Koychev, P Kurupati, X Li, E Lukaschuk, K McGlynn, H McShane, C Megson, K Motohashi, S Neubauer, D Nicoll, G Ogg, E Pacpaco, M Pavlides, Y Peng, N Petousi, J Propescu, N Rahman, B Raman, M J Rowland, K Saunders, M Sharpe, N Talbot, E Tunnicliffe |
| **Royal Brompton and Harefield Clinical Group, Guy’s and St Thomas’ NHS Foundation Trust.** |
| W D-C Man (PI), B Patel (PI), R E Barker, D Cristiano, N Dormand, M Gummadi, S Kon, K Liyanage, C M Nolan, S Patel, O Polgar, P Shah, S J Singh, J A Walsh |
| **Royal Free London NHS Foundation Trust** |
| J Hurst (PI), H Jarvis (PI), S Mandal (PI), S Ahmad, S Brill, L Lim, D Matila, O Olaosebikan, C Singh |
| **Royal Papworth Hospital NHS Foundation Trust** |
| M Toshner (PI), H Baxendale, L Garner, C Johnson, J Mackie, A Michael, J Pack, K Paques, H Parfrey, J Parmar |
| **Salford Royal NHS Foundation Trust** |
| N Diar Bakerly (PI), P Dark, D Evans, E Hardy, A Harvey, D Holgate, S Knight, N Mairs, N Majeed, L McMorrow, J Oxton, J Pendlebury, C Summersgill, R Ugwuoke, S Whittaker |
| **Salisbury NHS Foundation Trust** |
| W Matimba-Mupaya (PI), S Strong-Sheldrake |
| **Sheffield Teaching NHS Foundation Trust & University of Sheffield** |
| S L Rowland-Jones (PI), A A R Thompson (Co PI), J Bagshaw, M Begum, K Birchall, R Butcher, H Carborn, F Chan, K Chapman, Y Cheng, L Chetham, C Clark, Z Coburn, J Cole, M Dixon, A Fairman, J Finnigan, L Finnigan, H Foot, D Foote, A Ford, R Gregory, K Harrington, L Haslam, L Hesselden, J Hockridge, A Holbourn, B Holroyd-Hind, L Holt, A Howell, E Hurditch, F Ilyas, C Jarman, A Lawrie, E Lee, J-H Lee, R Lenagh, A Lye, I Macharia, M Marshall, A Mbuyisa, J McNeill, S Megson, J Meiring, L Milner, S Misra, H Newell, T Newman, C Norman, L Nwafor, D Pattenadk, M Plowright, J Porter, P Ravencroft, C Roddis, J Rodger, P Saunders, J Sidebottom, J Smith, L Smith, N Steele, G Stephens, R Stimpson, B Thamu, N Tinker, K Turner, H Turton, P Wade, S Walker, J Watson, I Wilson, A Zawia |
| **St George’s University Hospitals NHS Foundation Trust** |
| R Aul (PI), M Ali, A Dunleavy (PI), D Forton, N Msimanga, M Mencias, T Samakomva, S Siddique, J Teixeira, V Tavoukjian |
| **Sherwood Forest Hospitals NHS Foundation Trust** |
| J Hutchinson (PI), L Allsop, K Bennett, P Buckley, M Flynn, M Gill, C Goodwin, M Greatorex, H Gregory, C Heeley, L Holloway, M Holmes, J Kirk, W Lovegrove, TA Sewell, S Shelton, D Sissons, K Slack, S Smith, D Sowter, S Turner, V Whitworth, I Wynter |
| **Shropshire Community Health NHS Trust** |
| L Warburton (PI), S Painter, J Tomlinson |
| **Somerset NHS Foundation Trust** |
| C Vickers (PI), T Wainwright, D Redwood, J Tilley, S Palmer |
| **Swansea Bay University Health Board** |
| G A Davies (PI), L Connor, A Cook, T Rees, F Thaivalappil, C Thomas |
| **Tameside and Glossop Integrated Care NHS Foundation** |
| A Butt (PI), M Coulding, H Jones, S Kilroy, J McCormick, J McIntosh, H Savill, V Turner, J Vere |
| **The Great Western Hospital Foundation Trust** |
| E Fraile (PI), J Ugoji |
| **The Hillingdon Hospitals NHS Foundation Trust** |
| S S Kon (PI), H Lota, G Landers, M Nasseri, S Portukhay |
| **The Rotherham NHS Foundation Trust** |
| A Hormis (PI), A Daniels, J Ingham, L Zeidan |
| **United Lincolnshire Hospitals NHS Trust** |
| M Chablani (PI), L Osborne |
| **University College London Hospital & University College London** |
| M Marks (PI), J S Brown (PI), N Ahwireng, B Bang, D Basire, R C Chambers, A Checkley, R Evans, M Heightman, T Hillman, J Hurst, J Jacob, S Janes, R Jastrub, M Lipman, S Logan, D Lomas, M Merida Morillas, A Pakzad, H Plant, J C Porter, K Roy, E Wall, B Williams, M Xu |
| **University Hospital Birmingham NHS Foundation Trust & University of Birmingham** |
| D Parekh (PI), N Ahmad Haider, C Atkin, R Baggott, M Bates, A Botkai, A Casey, B Cooper, J Dasgin, K Draxlbauer, N Gautam, J Hazeldine, T Hiwot, S Holden, K Isaacs, T Jackson, S Johnson, V Kamwa, D Lewis, J M Lord, S Madathil, C McGhee, K Mcgee, A Neal, A Newton Cox, J Nyaboko, D Parekh, Z Peterkin, H Qureshi, B Rangelov, L Ratcliffe, E Sapey, J Short, T Soulsby, J Stockley, Z Suleiman, T Thompson, M Ventura, S Walder, C Welch, D Wilson, S Yasmin, K P Yip |
| **University Hospitals of Derby and Burton** |
| P Beckett (PI) C Dickens, U Nanda |
| **University Hospitals of Leicester NHS Trust & University of Leicester** |
| C E Brightling (CI), R A Evans (PI), M Aljaroof, N Armstrong, H Arnold, H Aung, M Bakali, M Bakau, M Baldwin, M Bingham, M Bourne, C Bourne, N Brunskill, P Cairns, L Carr, A Charalambou, C Christie, M J Davies, S Diver, S Edwards, C Edwardson, O Elneima, H Evans, J Finch, S Glover, N Goodman, B Gootpu, N J Greening, K Hadley, P Haldar, B Hargadon, V C Harris, L Houchen-Wolloff, W Ibrahim, L Ingram, K Khunti, A Lea, D Lee, G P McCann, H J C McAuley, P McCourt, T Mcnally, G Mills, A Moss, W Monteiro, M Pareek, S Parker, A Rowland, A Prickett, I N Qureshi, R Russell, N Samani, M Sereno, M Sharma, A Shikotra, S Siddiqui, A Singapuri, S J Singh, J Skeemer, M Soares, E Stringer, T Thornton, M Tobin, E Turner, L V Wain, T J C Ward, F Woodhead, J Wormleighton, T Yates, A Yousuf |
| **University Hospital Southampton NHS Foundation Trust & University of Southampton** |
| M G Jones (PI), C Childs, R Djukanovic, S Fletcher, M Harvey, E Marouzet, B Marshall, R Samuel, T Sass, T Wallis, H Wheeler |
| **Whittington Health NHS** |
| R Dharmagunawardena (PI), E Bright, P Crisp, M Stern |
| **Wirral University Teaching Hospital** |
| A Wight (PI), L Bailey, A Reddington |
| **Wrightington Wigan and Leigh NHS trust** |
| A Ashish (PI), J Cooper, E Robinson |
| **Yeovil District Hospital NHS Foundation Trust** |
| A Broadley (PI) |
| **York & Scarborough NHS Foundation Trust** |
| K Howard (PI), L Barman, C Brookes, K Elliott. L Griffiths, Z Guy, D Ionita, H Redfearn, C Sarginson, A Turnbull |
| **Health and Care Research Wales** |
| Y Ellis |
| **London School of Hygiene & Tropical Medicine (LSHTM)** |
| M Marks, A Briggs |
| **NIHR Office for Clinical Research Infrastructure** |
| K Holmes |
| **Patient Public Involvement Leads** |
| Asthma UK and British Lung Foundation Partnership - K Poinasamy, S Walker |
| **Royal Surrey NHS Foundation Trust** |
| M Halling-Brown |
| **South London and Maudsley NHS Foundation Trust & Kings College London** |
| G Breen, M Hotopf |
| **Swansea University & Swansea Welsh Network** |
| K Lewis, N Williams |

**References to support supplementary material**

[1] Benchimol EI, Smeeth L, Guttmann A, Harron K, Moher D, Petersen I, et al. The REporting of studies Conducted using Observational Routinely-collected health Data (RECORD) Statement. PLOS Medicine. 2015;12:e1001885.

[2] Davies MJ, Gray LJ, Troughton J, Gray A, Tuomilehto J, Farooqi A, et al. A community based primary prevention programme for type 2 diabetes integrating identification and lifestyle intervention for prevention: the Let's Prevent Diabetes cluster randomised controlled trial. Preventive medicine. 2016;84:48-56.

[3] Sampson M, Clark A, Bachmann M, Garner N, Irvine L, Howe A, et al. Lifestyle intervention with or without lay volunteers to prevent type 2 diabetes in people with impaired fasting glucose and/or nondiabetic hyperglycemia: a randomized clinical trial. JAMA Intern Med. 2021;181:168-78.

[4] World Health Organisation. Definition, diagnosis and classification of diabetes mellitus and its complications. Report of a WHO consultation. Part 1: Diabetes and classification of diabetes melllitus. Geneva: WHO; 1999.

[5] World Health Organisation. Use of glycated haemoglobin (HbA1c) in the diagnosis of diabetes mellitus. Geneva: WHO; 2011.

[6] Page MJ, McKenzie JE, Bossuyt PM, Boutron I, Hoffmann TC, Mulrow CD, et al. The PRISMA 2020 statement: an updated guideline for reporting systematic reviews. BMJ. 2021;372:n71.
